# Investigating the neural effects of typicality and predictability for face and object stimuli

**DOI:** 10.1371/journal.pone.0293781

**Published:** 2024-05-22

**Authors:** Linda Ficco, Chenglin Li, Jürgen M. Kaufmann, Stefan R. Schweinberger, Gyula Z. Kovács

**Affiliations:** 1 Department of General Psychology and Cognitive Neuroscience, Friedrich Schiller University, Jena, Germany; 2 Department of Biological Psychology and Cognitive Neurosciences, Friedrich Schiller University, Jena, Germany; 3 International Max-Planck Research School for the Science of Human History, Jena, Germany; 4 School of Psychology, Zhejiang Normal University, Jinhua, China; Medical University of Vienna: Medizinische Universitat Wien, AUSTRIA

## Abstract

The brain calibrates itself based on the past stimulus diet, which makes frequently observed stimuli appear as typical (as opposed to uncommon stimuli, which appear as distinctive). Based on predictive processing theory, the brain should be more “prepared” for typical exemplars, because these contain information that has been encountered frequently, allowing it to economically represent items of that category. Thus, one could ask whether predictability and typicality of visual stimuli interact, or rather act in an additive manner. We adapted the design by Egner and colleagues (2010), who used cues to induce expectations about stimulus category (face vs. chair) occurrence during an orthogonal inversion detection task. We measured BOLD responses with fMRI in 35 participants. First, distinctive stimuli always elicited stronger responses than typical ones in all ROIs, and our whole-brain directional contrasts for the effects of typicality and distinctiveness converge with previous findings. Second and importantly, we could not replicate the interaction between category and predictability reported by Egner et al. (2010), which casts doubt on whether cueing designs are ideal to elicit reliable predictability effects. Third, likely as a consequence of the lack of predictability effects, we found no interaction between predictability and typicality in any of the four tested regions (bilateral fusiform face areas, lateral occipital complexes) when considering both categories, nor in the whole brain. We discuss the issue of replicability in neuroscience and sketch an agenda for how future studies might address the same question.

## 1. Introduction

### 1.1 Perceptual spaces in visual perception

If someone asks you to imagine the picture of a chair, chances are that it would be made of wood, have four legs, be rather symmetrical, have a simple backrest and maybe a pillow. Within any visual category, some exemplars are more representative than others, and are perceived as “typical” [1]. The perception of typicality depends on the fact that exemplars are not represented independently in our mind (and brain). Any chair exemplar is remembered and processed in relation to other similar-looking items, seen in similar contexts, with the same function, size, material, and so on. Perceptual categories, such as “chairs”, can be thought of as low-dimensional perceptual spaces of various exemplars [2–4], with minimal differences and maximal similarities across them ([1], p. 491).

In such models, typical exemplars share most features with each other [5], have more average features [6], stand out of the category less [7], and summarize its dimensions best [5, 8]. Importantly, the perception of typicality is flexible: it is influenced by the current perceptual diet of the observer [9] and its stability depends on the duration and amount of previous exposure (see [1] for a discussion).

Faces are thought to be represented in such space [6, 10, 11]. We are exposed to thousands of faces throughout our life [12] and develop expertise for this category [13, 14]. Our face diet shapes the prototype in reference to which new faces are encoded constantly [15–18], and people with allegedly comparable face spaces tend to form coherent typicality judgements about the same faces [19].

A long-standing debate exists regarding different face-space models [11, 20, 21]. In exemplar-based models faces are encoded based on their similarity, at relative distances from each other [6, 22]. Conversely, in norm-based models, faces are encoded with respect to a weighted average of all seen faces [23]. Notably, norm-based coding appears to extend beyond facial stimuli to non-face objects as well [24–27]. When comparing the two models explicitly, the norm-based model seems to account for a number of findings–e.g., responses in the fusiform face area–better than the exemplar-based model [28]. However, note that other works comparing these two models, and using visual stimuli for which participants have acquired expertise (e.g., faces, real objects and abstract shapes), seem to find support for both [21, 24, 29, 30].

### 1.2 Behavioural and neural effects of typicality

Despite different existing operationalizations of typicality (e.g., different kinds of ratings, via morphing, etc…), its effects are relatively uncontroversial: Neural and behavioural data generally show that typical stimuli are easier to *process* (that is, performing mental operations, such as detection or classification, on them) but harder to *encode* (i.e., store in a mental representation). Behaviourally, typical stimuli are categorized faster and more accurately than distinctive ones [24, 25, 31–34]. Moreover, typical stimuli are detected faster than distinctive ones [35], as suggested by detection advantages for own-“race” (typical), as compared to other-“race” faces [6, 36]. However, typicality hampers the recognition of specific exemplars: Distinctive faces are recognized more accurately [37–47] and faster [34, 37–39, 46 but see 48] than typical faces. This is possibly due to poorer encoding of typical stimuli [49], which are more similar to each other and can be confused more easily, leading to false positive answers due to impaired pattern separation processes [50].

Several neuroimaging techniques have been used to map perceptual spaces (reviewed in [51]. Neuroimaging findings suggest that typical stimuli require i) smaller brain metabolic changes and ii) reduced configural processing, as compared to distinctive stimuli. Specifically, fMRI studies report increased brain responses to distinctive stimuli, both in the visual [28, 52, 53] and in the auditory modality [54, 55]. In electrophysiological studies using event-related potentials (ERPs), face typicality consistently affects the amplitude of the occipito-temporal P200 component, with larger amplitudes for typical, as compared to distinctive stimuli [10, 56–58]. Object typicality can affect occipito-temporal ERPs even at latencies < 200 ms [59]; studies using objects or animal images report larger P200 in frontal regions to distinctive as compared to typical objects [31] and shorter latencies of the P300 component for typical stimuli [60]. These findings parallel reaction time data [60] and could indicate that typical stimuli require less attention and lower feature encoding [61].

Importantly, and although it should be kept in mind that the precise definition of typicality may vary across individual studies, neural and behavioural typicality effects appear to be related, and their relationship cannot be explained by the mere physical similarity of stimuli [52].

### 1.3 Typicality effects under the predictive coding framework: The current study

Visual perception not only capitalizes on dimensionality reduction (as discussed above), but also on the prediction of upcoming sensory inputs [62]. According to the theory of predictive processing [63–65], the brain attempts to predict the input received from lower processing levels, by generating an internal model of the input at each level of stimulus processing (be it single neurons, neural populations, or network hubs) [66, 67]. The degree to which this internal model differs from the incoming input represents the prediction error (i.e., the mismatch between actual and predicted input; [68]), which is then transferred to higher levels [67]. These recursive model-error comparison loops tend to minimize prediction error [69], and ultimately allow the individual to generate and maintain the most accurate and up-to-date models of the sensory world. In other words, perception is not a passive process, but an active attempt of the brain to best guess the latent causes of a sensory input, informed by prior knowledge [70, 71].

Visual prototypes seem to be just this: representations “summarizing" stimuli from a particular category best, based on those stimuli we have frequently experienced in the past. Potentially, this theory elegantly explains the neural and behavioural effects of stimulus typicality as well: typical stimuli elicit less prediction error than distinctive ones when compared to a prototype, thus leading to faster detection and categorization, and lower neural responses in regions processing stimulus configuration and category. However, to date systematic research on the combined effects of predictability and typicality is missing. To address this important theoretical and empirical gap, one should test to which degree typicality and predictability *interact*, or whether they act in an *additive manner*. An interaction between predictability and typicality would imply a common neural mechanism underlying both effects, whereas an additive effect would be more consistent with separable mechanisms to compute stimulus probability and typicality (a logic similar to that used in [72, 73]. Accordingly, if two processes are independent, the expression of one effect should not change for different levels of the other (i.e., the two effects should just add up). Instead, if there is an additional increase in one effect for one level of the other that is not explained by a simple addition of the two effects, one can infer an (at least partial) dependency of the mechanisms underlying the two effects.

To test this, we presented face or chair stimuli, preceded by a cue which signalled to participants the probability of the occurrence of a particular stimulus category (“predictability” here thus indicates expected temporal association between a stimulus category and a cue preceding the stimulus). Importantly, orthogonal to this predictability modulation, we also manipulated stimulus typicality by presenting typical and distinctive stimulus exemplars (so, typicality here refers to the distance of each stimulus from an average exemplar of the same category–see Section 2.2). We replicated a design that had produced an interaction between category and predictability in a previous study [74], and that could allow us to elegantly test the presence of an interaction. Another important goal of the present work was to assess the replicability of this effect, especially since a recent, large study [75] has shown that it is challenging to produce neural predictability effects through cueing, despite effective behavioural training.

## 2. Materials and methods

### 2.1 Participants

We recruited thirty-five adult participants (22 females, one diverse; mean age = 24.4 years; *SD* = 4.0 years; two left-handed, one ambidextrous), with normal or corrected-to-normal vision and with no reported neurological condition (one participant reported a diagnosis of Asperger Syndrome, data of this participant were retained in the analysis). The measurements were performed between May and August 2022. The main experimenter had access to identifying information from participants and took care of making their data anonymous for the analyses. We ensured that each of them had more than 10 years of exposure to Caucasian/European/White faces. The sample size was determined by a power analysis with the R package *Superpower* [76]. We calculated the number of participants necessary to detect a medium effect size (f = 0.25) for the interaction of cue-induced predictability and category, to achieve a power of 0.80 at the standard .05 alpha error probability. This was calculated based on the mean beta estimates in different conditions reported in Fig 3 of [74]. We chose this effect for our power analysis because we had no expectations about the size of the interaction of interest, so we preferred to calibrate our sample size to target a known effect. This led to a sample size that nearly doubled the sample reported in [74], who recruited 17 participants. Participants could choose between monetary compensation and a small 3D model of their own brain for their participation. Before the experiment, all participants received information about the experimental procedures and provided their written informed consent. The models contributing to our 3D-face database have provided their written consent to the experimental use of their face stimuli. The models shown in this article had specifically agreed on being displayed in scientific publications. The ethics committee of the Friedrich Schiller University Jena approved the experimental protocol (Reg. No. FSV 22/086), and the study was conducted in accordance with the guidelines of the Declaration of Helsinki. The pre-registration for this study can be found at: https://osf.io/axstg/?view_only=13f0e8794885499d805e6649670b2f13.

### 2.2 Stimuli

In addition to images of faces, we chose images of chairs as control stimuli, as they show approximate vertical symmetry and a clear upright direction. Both these features seem to be important in norm-based coding of objects [77]. As cues we used geometrical shapes of different colours (a green, a blue, and a yellow frame, with similar area, brightness, and saturation levels). All stimuli were presented by Psychtoolbox-3 (MATLAB-based; [78, 79]).

We manipulated perceived typicality of faces and chairs as follows. For faces, we followed previous work as regards the stimulus database and the manipulation approach [10, 80–83]. In detail, we created three-dimensional face stimuli using DI3Dcapture™ (Dimensional Imaging, Glasgow, UK). Each face had been photographed by four cameras simultaneously, and the images were interpolated to create a three-dimensional (3D) object. Using DI3Dview™, we generated caricatures and anti-caricatures (both in shape and texture), by extrapolating each individual veridical 3D file with the morphing tool with respect to a gender-matched average. Finally, because the 3D camera system makes it possible to extract images from various viewing angles, we produced our stimuli by systematically tilting the individual faces according to a set of ten viewpoints (see [80] for a more detailed description of this procedure). Based on a previous rating study, we selected 24 face identities (half female and half male). We subjected each face to both caricaturing (shifting both face shape and texture + 0.33 units on the face trajectory, *opposite to* the face norm) and anti-caricaturing (shifting both face shape and texture + 0.33 units on the face trajectory, *in the direction of* the face norm). For each participant, we selected only one caricaturing version per identity (counterbalanced across participants), leading to 12 caricatured (distinctive) and 12 anti-caricatured (typical) identities presented to each participant. Throughout the manuscript we use the term “typical” to refer both to faces morphed towards the average and to chairs rated as typical. Conversely, the term “distinctive” refers both to faces morphed in the opposite direction with respect to the average and to chairs rated as distinctive. In contrast, “anti-caricatured” and “caricatured” always refer to the procedure applied to make faces typical and distinctive, respectively. Since we wanted to avoid image-dependent identity perception (i.e., if the same picture of Identity 4 were shown throughout the experiment), we produced 10 different images for each identity. We used Adobe Photoshop™ to tilt each 3D-face on 10 pre-set angles, resulting in 240 2D stimuli in total for participant for each task run.

Colour images of chairs were downloaded from the Internet and were subjected to standard pre-processing to ensure similar image size, quality, and a homogeneous background. No chair contained written text or recognizable logos. To make the chairs comparable to the face images, we used 10 images per chair, each showing the image at a comparable set of angles. We manipulated typicality by selecting the 12 most typical and the 12 most distinctive chair images, as rated by a separate group of 10 participants (Cronbach’s alpha = 0.991).

**Fig 1:**
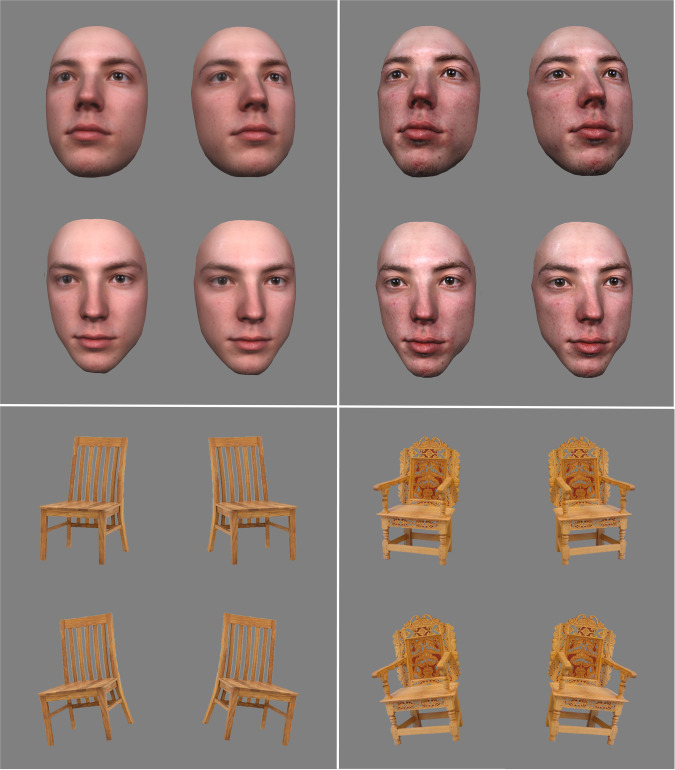
Stimulus examples. Comparison of the same face identity in its caricatured and anti-caricatured version. Note that each identity was shown to each participant in one of the two versions only. Left: a typical (anti-caricatured) face and a typical chair. Right: a distinctive (caricatured) face and a distinctive chair.

Both face and chair stimuli were presented in black and white, to avoid confounding effects due to the wider range of colours of chairs, as compared to faces. See Fig 1 for examples of face and chair stimuli.

### 2.3 Experimental design and procedure

**Fig 2:**
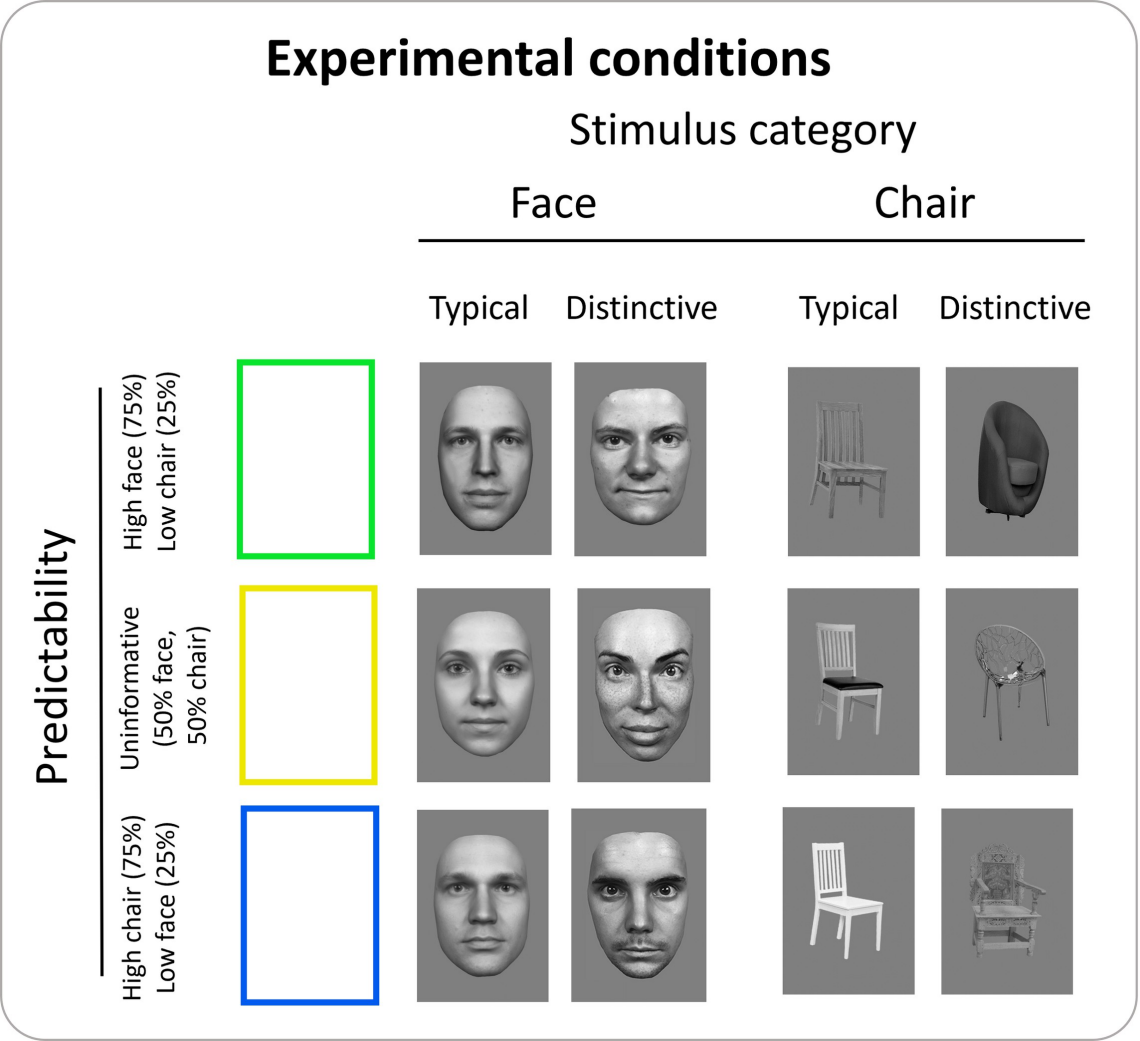
Graphical representation of the experimental conditions.

To manipulate predictability of stimulus category (face or chair), a green frame preceded a face in 75% of the trials, and a chair in the remaining 25% of trials. Conversely, a blue frame preceded a chair in 75% of the trials and a face in the remaining 25%. As suggested by [84] and following [74], we included a neutral, uninformative condition, whereby a yellow frame preceded faces or chairs equally often (50% of the trials). Experimental conditions are shown in Fig 2. Throughout the task, participants saw 24 identities (12 faces + 12 chairs), in 10 viewpoints each. Participants performed four task runs in total (two participants performed only three runs due to technical issues). The order of trials within each experimental run was randomized. Each trial started with a fixation cross, which also separated trials (2000–4000 ms, plus a blank if participants responded, to ensure that the duration of one trial is an integer multiple of our repetition time; TR = 2), followed by a cue (250 ms), and a target stimulus (750 ms). To ensure participants’ attentional focus, in 10% of trials the stimulus was inverted (but otherwise followed the same cue contingencies as used for upright stimuli), and participants performed an orthogonal detection task by pressing a key to inverted stimuli with their right index finger on an MRT-compatible keyboard. Our inter-trial intervals (2, 3, or 4 s) were randomized [74]. The experiment included a total of 528 trials, divided into 4 runs of 132 trials each (39 usable, non-target trials per condition in each run). Target trials were excluded from analyses. Participants were informed about the cue contingencies, but we specified that these were not task relevant. We did so with the aim of replicating the design by [74], who instructed their participants in a similar way. Before completing the task, participants were familiarized with the procedure and the contingencies through a short practice session (~2.5 minutes) outside the scanner.

After completing the fMRI measurement, participants completed a short questionnaire in which they reported the degree to which they experienced any difficulties during scanning, paid attention to the contingencies, and thought the faces and chairs looked typical or distinctive (cf. S1 File).

### 2.4 Imaging parameters and data analysis

We used a 3-Tesla MR scanner (Siemens MAGNETOM Prisma fit, Erlangen, Germany) with a 20-channel head coil to record BOLD responses to our manipulations. T1-weighted high-resolution 3D anatomical images were acquired with an MP-RAGE sequence (192 slices; TR = 2300 ms; TE = 3.03 s; flip angle = 90°; 1 mm isotropic voxel size). As for the four functional runs, T2*-weighted fMRI-images were collected with a multi-band EPI sequence (MB acceleration factor = 8) under the following parameters: 34 slices; FOV = 204 x 204 mm2; TR = 2000 ms; TE = 30 ms; flip angle = 90°; 2 mm isotropic voxel size. Similarly to a previous study [85], we used SPM12 (Welcome Department of Imaging Neuroscience, London, UK), based on MATLAB version R2020a, to pre-process data.

The experiment was based on an event-related design. Functional images were slice-timed, realigned (the structural image was realigned to a mean image computed from the functional series, and co-registered to structural scans). We then normalized the images to the MNI-152 space, resampled to 2 x 2 x 2 mm resolution, and spatially smoothed using an 8-mm Gaussian kernel. The convolution of a reference hemodynamic response function (HRF) with box cars, representing the onsets and durations of the experimental conditions, was used to define the regressors for a general linear model analysis of the data. For each of the experimental conditions we modelled the HRF, synchronised to the onset of the trial. Low frequency components were excluded from the model using a high-pass filter with 128 s cut-off. Variance which could be explained by the previous scans was excluded using an autoregressive AR(1) model, and movement-related variance was accounted for by the spatial parameters resulting from the realignment procedure. The resulting regressors were fitted to the observed functional time series. For the random effects analysis, the contrast estimates entered a simple t-test or an F-test at the second level (see S3 Table for the regressor weights in the whole-brain analysis). Results were thresholded at *p* < .05 (voxelwise, uncorrected), with a cluster correction for multiple comparisons at FWE < .05. For visualization, the thresholded t-images were superimposed onto a standard template available on MRIcroGL (https://www.nitrc.org/projects/mricrogl/). We extracted the peaks of maximal activation and their respective anatomical labels using the Anatomy Toolbox implemented in [86] and the Talairach Daemon Client [87, 88].

Additionally, participants performed a 6-minute functional localizer task, so that we could isolate regions of interest (ROIs). Faces, everyday objects, and Fourier-randomized noise images were presented (4 Hz; 230 ms exposition time; 20 ms ISI) in blocks of 10 s, interrupted by breaks of 10 s and repeated five times. Each block included 40 images (size: 400 x 400 pixels with a grey background). Participants were instructed to observe the images and maintain their attentional focus on the screen. The fusiform face area (FFA; [89, 90]) and the lateral occipital complex (LOC; [91, 92]) were isolated. The object-selective area LO [91, 93] corresponded to the posterior dorsal portion of the LOC [94]). We determined the location of the FFA in individual participants by contrasting face and object blocks (or face and noise blocks, if the former led to no significant voxels) and established as the local maximum from the t-maps with a threshold of *p*_*FWE*_< 0.05 on the single-subject level. A similar approach was taken to locate the LOC (objects > noise blocks, t-maps with a threshold of *p*_*FWE*_< 0.05), on the single-subject level. We report the individual MNI coordinates in S1 Table, and report the average (± SD) here: right FFA (*x*_*M*_ = 40.6, *x*_*SD*_ = ± 4.7; *y*_*M*_ = -52.9, *y*_*SD*_ = ± 7.7; *z*_*M*_ = -18.6, *z*_*SD*_ = ± 3.9), left FFA (*x*_*M*_ = -38.9, *x*_*SD*_ = ± 3.5; *y*_*M*_ = -52.4, *y*_*SD*_ = ± 6.9; *z*_*M*_ = -18.9, *z*_*SD*_ = ± 4.0), right LOC (*x*_*M*_ = 40.1, *x*_*SD*_ = ± 4.6; *y*_*M*_ = -76.1, *y*_*SD*_ = ± 8.2; *z*_*M*_ = -3.6, *z*_*SD*_ = ± 6.4), left LOC (*x*_*M*_ = -43.1, *x*_*SD*_ = ± 5.1; *y*_*M*_ = -78.3, *y*_*SD*_ = ± 6.6; *z*_*M*_ = -3.5, *z*_*SD*_ = ± 5.7). Areas matching anatomical criteria were considered as their appropriate equivalents on the single-subject level. A time-series of the mean voxel value within a 4 mm radius sphere around the local peak was extracted from our event-related sessions using finite impulse response (FIR) models [95] for each area and participant separately. As for the main task analysis, we convolved our data with a reference HRF using boxcars, to define the regressors for a general linear model using MarsBaR 0.44 toolbox for SPM 12 [96]. The peak BOLD values (corresponding to the third TR post-stimulus onset) were extracted from the four event-related runs while trials with inverted stimuli were excluded from the analysis. We analysed only upright trials using repeated-measures ANOVAs in each ROI with cue-induced Predictability (3, high-face expectation, uninformative, high-chair expectation), Category (2, faces and chairs) and Typicality (2, typical and distinctive) as within-subject factors. We were especially interested in the main effects of typicality (both overall and separate by category), the interaction between category and predictability (replication of [74]’s results) and, critically, the interaction between typicality and predictability. These analyses were performed on JASP (Version 0.16.3). All multiple comparisons of post-hoc tests were corrected using Holm’s method. All data and scripts are available on OSF (view-only link for peer review: https://osf.io/x5cyq/?view_only=07d874205ea446b0b54a098542cb93b3).

## 3. Results

### 3.1 Behavioural results

Participant’s accuracy was close to ceiling (*M* = 0.99 proportion correct responses, *SD* = 0.01, response time: *M* = 543 ms, *SD* = 77 ms). This suggests that they attended to the task well, and that the task was easy to perform. Due to the small number of inverted trials, we did not perform statistical analysis, but note that numerically, participants were faster for the detection of inverted faces than for inverted chairs, *M* = 525 vs. 553 ms, respectively. All participants confirmed that experimental faces were similar to those encountered throughout their life. On average, participants reported only relatively low attention to cue contingencies (*M* = 0.77 on a scale from 0 to 3; see S2 Table for details) and we detected no effects of predictability on RT (*p* = .1692). Note that participants were instructed that cue contingencies were irrelevant to task completion. Finally, the presence of a typicality manipulation was subjectively noticed for chairs more often than for faces, *M* = 20/35 vs. 5/35 participants, respectively. We exploratively investigated behavioural effects of typicality, but found no differences between typical and distinctive stimuli, neither for RTs (*p* = .163) nor for accuracy (*F*(1, 416) = 1.237, *p* = .267).

### 3.2 Neuroimaging results

#### 3.2.1 Regions-of-interest

**Fig 3:**
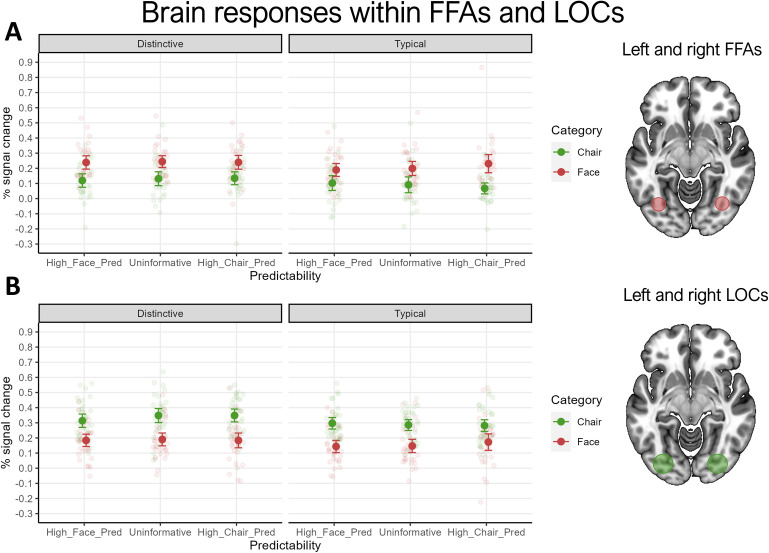
Results of the ROI analysis. Brain responses to typical and distinctive stimuli of the two categories to facilitate the comparison with Fig 3 in [74]. High_Face_Pred: cue that highly predicts a face; High_Chair_Pred: cue that highly predicts a chair; Uninformative: cue that predicts either category equally; panel A: brain responses to typical and distinctive stimuli in both left and right FFAs averaged; panel B: brain responses to typical and distinctive stimuli in both left and right LOCs averaged. For a figure depicting brain responses in individual ROIs, see S1 Fig. ROIs shown on the right for illustration purposes only; the average locations of the individually defined ROIs are indicated in Section 2.4. Error bars represent 95% confidence intervals.

We found prominent effects of Category and Typicality in all our ROIs (Fig 3). Here we report the average between left and right hemisphere, see the S1 File for results for the individual ROIs. Considering all participants, from a total of 12600 non-target trials (counted considering all participants together), only 32 were false alarms. Since this number is low, and we did not have reason to expect that these would affect any of our experimental conditions differently, we did not remove them from the brain data analysis. In the FFA, the main effect of Category was significant (*F*(1,28) = 29.058, *p* < .001, $\eta_{p}^{2}$ = 0.509), with faces eliciting larger responses than chairs (*p =* .001). The effect of Category was also significant in the LOC (*F*(1,33) = 103.146, *p* < .001, $\eta_{p}^{2}$ = 0.763), where chairs elicited larger responses than faces. Considering that these regions were selected based on the functional localizer and extensive previous literature, these results are expected, and can be seen as an indicator of data quality. More interesting are the effects of Typicality over the four ROIs (all *p*s < .001), which were at times comparably sized as those of Category (e.g., right FFA, $\eta_{p}^{2}$ = 0.612; range $\eta_{p}^{2}\text{s}$ = 0.257–0.612). The effect of Typicality was significant and in the same direction, even when considering only the stimulus category for which each ROI is specialized (i.e., faces for FFAs and chairs for LOCs; all *ps*_*Holm*_< .005; see S1 Fig). Remarkably, the hypothesized interactions between stimulus Typicality and Predictability failed to reach significance (cf. Table 1 for details). We additionally analysed this interaction when only considering the preferred stimulus category of the ROIs (faces for FFA, chairs for LOC). Again, the interactions did not reach significance, although in the LOC we noted a trend (*p* = .081), with distinctive chairs eliciting larger responses than typical chairs especially in the uninformative condition. In line with [74], we found no main effect of Predictability in any ROI. We also could not replicate the interaction between Category and Predictability reported by [74], and no other interactions reached significance. We additionally report the Bayes Factor for the main models we tested in the FFAs and the LOCs. In both cases, the model with the highest evidence in favour of the alternative hypothesis was that including the two main effects of Category and Typicality but not their interaction (*BF*_10_ = 1.000 in the FFAs, and *BF*_10_ = 1.000 in the LOCs). These values can be interpreted as weak evidence in favour of the described model [97]. Additionally, in both cases the model with the strongest evidence in favour of the null hypothesis is the one including solely the main effect of Predictability (*BF*_10_ = 5.363^−7^ in the FFAs, and *BF*_10_ = 7.147^−13^ in the LOCs). The analysis for our model of interest, including the main effects of Category, Typicality and Predictability, plus the interactions Predictability*Typicality, returned strong support in favour of the null hypothesis both in the FFAs (*BF*_10_ = 0.005) and in the LOCs (*BF*_10_ = 0.028) [97]. Finally, the model including Category, Predictability and their interaction in the FFAs, which would correspond to the effect reported in [74], received strong evidence in favour of the null hypothesis (*BF*_10_ = 2.234^−4^). Overall, these analyses are consistent with the effects calculated under a frequentist framework—although the evidence in favour of our two main effects remains weak and points to the absence of our interaction of interest.

**Table 1:** Within-subjects ANOVAs results and contrast weights.

**Left FFA (N = 21)**						
*Effect*	*Sum of Squares (residuals)*	*Df (residuals)*	*Mean Square (residuals)*	*F*	*p*	* $\eta_{p}^{2}$ *
Predictability	0.005^a^ (0.439)	2^a^ (40)	0.003^a^ (0.011)	0.24^a^	.792^a^	.012^a^
**Category**	**0.977 (1.180)**	**1 (20)**	**0.977 (0.059)**	**16.56**	**< .001**	**.453**
**Typicality**	**0.077 (0.156)**	**1 (20)**	**0.077 (0.008)**	**9.82**	**.005**	**.329**
Predictability * Category	0.029^a^ (0.354)	2^a^ (40)	0.014^a^ (0.009)	1.62^a^	.211^a^	.075^a^
Predictability * Typicality	0.004 (0.294)	2 (40)	0.002 (0.007)	0.25	.780	.012
Category * Typicality	0.217 (0.363)	1 (20)	0.217(0.018)	0.01	.929	.004
Predictability * Category * Typicality	0.030^a^ (0.647)	2^a^ (40)	0.015^a^ (0.016)	1.93^a^	.403^a^	.044^a^
**Right FFA (N = 27)**						
*Effect*	*Sum of Squares (residuals)*	*Df (residuals)*	*Mean Square (residuals)*	*F*	*p*	$\eta_{p}^{2}$
Predictability	0.016 (0.493)	2 (52)	0.008 (0.009)	0.87	.427	.032
**Category**	**0.880 (0.988)**	**1 (26)**	**0.880 (0.038)**	**23.16**	**< .001**	**.471**
**Typicality**	**0.180 (0.114)**	**1 (26)**	**0.180 (0.004)**	**40.97**	**< .001**	**.612**
Predictability * Category	0.009 (0.468)	2 (52)	0.005 (0.009)	0.52	.599	.020
Predictability * Typicality	0.003 (0.296)	2 (52)	0.001 (0.006)	0.23	.797	.009
Category * Typicality	0.006 (0.128)	1 (26)	0.006 (0.005)	1.27	.271	.046
Predictability * Category * Typicality	0.007 (0.382)	2 (52)	0.004 (0.007)	0.49	.614	.019
**Left LOC (N = 29)**						
*Effect*	*Sum of Squares (residuals)*	*Df (residuals)*	*Mean Square (residuals)*	*F*	*p*	$\eta_{p}^{2}$
Predictability	0.011 (0.482)	2 (56)	0.006 (0.009)	0.65	.529	.023
**Category**	**1.379 (0.525)**	**1 (28)**	**1.379 (0.019)**	**73.52**	**< .001**	**.724**
**Typicality**	**0.090 (0.261)**	**1 (28)**	**0.090 (0.009)**	**9.70**	**.004**	**.257**
Predictability * Category	**0.010 (0.357)**	**2 (56)**	0.005 (0.006)	0.80	.454	.028
Predictability * Typicality	0.002 (0.277)	2 (56)	0.001 (0.005)	0.24	.791	.008
Category * Typicality	0.017 (0.198)	1 (28)	0.017 (0.007)	2.47	.127	.081
Predictability * Category * Typicality	0.013 (0.549)	2 (56)	0.006 (0.010)	0.65	.526	.023
**Right LOC (N = 31)**						
*Effect*	*Sum of Squares (residuals)*	*Df (residuals)*	*Mean Square (residuals)*	*F*	*p*	$\eta_{p}^{2}$
Predictability	0.012 (0.588)	2 (60)	0.006 (0.010)	0.59	.557	.019
**Category**	**2.340 (1.101)**	**1 (30)**	**2.340 (0.037)**	**63.79**	**< .001**	**.680**
**Typicality**	**0.218 (0.222)**	**1 (30)**	**0.218 (0.007)**	**29.49**	**< .001**	**.496**
Predictability * Category	0.006 (0.411)	2 (60)	0.003 (0.007)	0.41	.666	.013
Predictability * Typicality	0.028 (0.387)	2 (60)	0.014 (0.006)	2.14	.126	.067
Category * Typicality	0.011 (0.123)	1 (30)	0.011 (0.004)	2.65	.114	.081
Predictability * Category * Typicality	0.019 (0.509)	2 (60)	0.010 (0.008)	1.12	.333	.036
**Average of left and right FFAs (N = 29)**						
*Effect*	*Sum of Squares (residuals)*	*Df (residuals)*	*Mean Square (residuals)*	*F*	*p*	$\eta_{p}^{2}$
Predictability	0.002 (0.453)	2 (56)	0.000 (0.008)	0.12	.890	.004
**Category**	**1.170 (1.128)**	**1 (28)**	**1.170 (0.040)**	**29.06**	**< .001**	**.509**
**Typicality**	**0.125 (0.153)**	**1 (28)**	**0.125 (0.005)**	**22.87**	**< .001**	**.450**
Predictability * Category	0.015 (0.334)	**2 (56)**	0.008 (0.008)	1.30	.281	.044
Predictability * Typicality	0.001 (0.285)	2 (56)	0.000 (0.005)	0.14	.867	.005
Category * Typicality	0.000 (0.204)	1 (28)	0.000 (0.007)	0.132	.719	.005
Predictability * Category * Typicality	0.031 (0.428)	2 (56)	0.016 (0.008)	2.04	.193	.068
**Average of left and right LOCs (N = 33)**						
*Effect*	*Sum of Squares (residuals)*	*Df (residuals)*	*Mean Square (residuals)*	*F*	*p*	$\eta_{p}^{2}$
Predictability	0.010 (0.496)	2 (64)	0.005 (0.008)	0.64	.529	.020
**Category**	**2.002 (0.621)**	**1 (32)**	**2.002 (0.019)**	**103.15**	**< .001**	**.763**
**Typicality**	**0.160 (0.211)**	**1 (32)**	**0.160 (0.007)**	**24.25**	**< .001**	**.431**
Predictability * Category	0.002 (0.353)	2 (64)	0.001 (0.006)	0.19	.825	.006
Predictability * Typicality	0.010 (0.322)	2 (64)	0.005 (0.005)	0.96	.387	.029
Category * Typicality	0.007 (0.187)	1 (32)	0.007 (0.006)	1.25	.273	.037
Predictability * Category * Typicality	0.026 (0.491)	2 (64)	0.013 (0.008)	1.69	.193	.050

*Within-subjects ANOVAs with Predictability (3 levels)*, *Category (2 levels) and Typicality (2 levels) as within-subjects factors*. *Results are presented both in each ROI singularly and in face- and object-responsive regions averaged across hemispheres*. *Significant effects are boldened*. *Sum of squares of type III are reported*. ^*a*^ *Mauchly’s test of sphericity indicates that the assumption of sphericity is violated (p <* .*05)*.

#### 3.2.2 Whole-brain analyses

**Fig 4:**
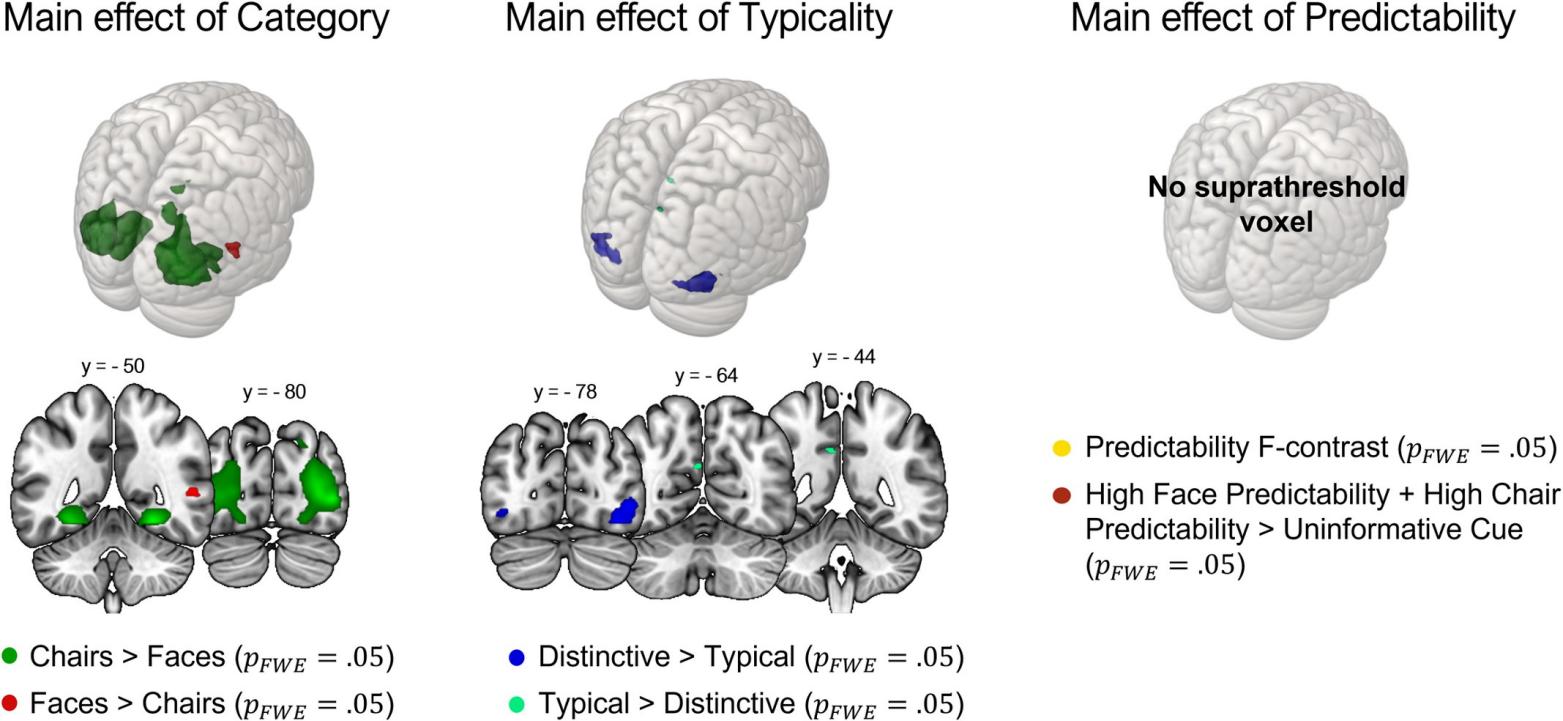
Whole brain results (MNI standard space). From left to right: directional t-contrasts of brain activation in response to faces > chairs (red) and chairs > faces /green); directional t-contrasts of brain activations in response to distinctive > typical stimuli (dark blue) and typical > distinctive (light green); non-directional F contrast assessing whether any of the predictability levels differs from the others (yellow) and directional t-contrast comparing the two conditions of high predictability to the uninformative condition (dark red). All contrasts are shown at an alpha level of .05, with a family-wise correction for multiple comparisons.

We investigated the main effects of Predictability (both by means of a non-directional F contrast and a directional t-contrast comparing high predictability conditions to the uninformative one), Typicality and Category. We also investigated the interaction of interest (Predictability * Typicality). The contrast faces > chairs revealed one cluster in the right superior temporal gyrus (rSTG), whereas the opposite contrast returned activation in two large bilateral clusters mainly encompassing the LOCs, the fusiform gyri, the inferior and middle occipital gyri, the lingual gyri, the precuneus, extending to the hippocampal cornu ammonis, and to the inferior and superior parietal lobules. The contrast typical > distinctive generated a small cluster in the left superior parietal lobe, while the opposite (distinctive > typical) revealed two symmetrical clusters which included the LOCs, the fusiform gyri and the ventral portions of V2. We report the same effect estimated in faces and chairs separately in S2 Fig. The main effect of Predictability resulted in no suprathreshold clusters (*p* < .05, FWE corrected), and relaxing the threshold to the conventionally accepted level of *p* < .001 (uncorrected) resulted in two small clusters in the right hemisphere, one in the anterior cingulate gyrus, and the other in the anterior insula. The directional contrast reflecting high predictability conditions compared to the uninformative one revealed no significant results (*p* < .05, FWE corrected). Setting a more lenient threshold at *p* < .0001, uncorrected resulted only in a three-voxel cluster in the right anterior cingulate gyrus which we report, but refrain from interpreting. The contrast relative to the interaction between Predictability and Typicality was not significant. The described effects are shown in Fig 4 and reported more in detail in Table 2.

**Table 2:** Local maxima of activation for the contrasts of interest at the whole brain level. Note that t- or F-values are provided for the peaks output by SPM12, whereas the other peaks have been located with the Anatomy Toolbox. For them all, the third column reports the anatomical location assigned via the Anatomy Toolbox. The raw output of the Anatomy Toolbox is provided as S1 File.

Contrast	Local maxima	Anatomical locations	Cluster size (voxels)
	MNI standard space	(Brodmann area)	
	x, y, z (t- or F-values)		
**Chairs > Faces (t-contrast) p > .05, FWE**			
Cluster 1	26, -52, -10 (13.95)	Right fusiform gyrus (BA 19)	2763
	42, -80, 4 (13.51)	Right middle occipital gyrus (BA 19)	
	32, -84, 14 (12.87)	Right middle occipital gyrus (BA 19)	
	22, -76, -10	Right lingual gyrus (BA 18)	
	18, -78, 44	Right precuneus (BA 7)	
	20, -76, 42	Right precuneus (BA 7)	
	48, -58, -6	Right middle occipital gyrus (BA 19)	
Cluster 2	-36, -88, 8 (13.81)	Left middle occipital gyrus (BA 19)	2542
	-30, -44, -12 (12.41)	Left fusiform gyrus (BA 37)	
	-32, -46, -10	Left fusiform gyrus (BA 37)	
	26, -58, -8	Right fusiform gyrus (BA 19)	
	-42, -78, -2	Left inferior occipital gyrus (BA 19)	
	-30, -74, 0	Left lingual gyrus (BA 19)	
	-44, -62, -4	Left middle temporal gyrus (BA 37)	
	-20, -76, -10	Left lingual gyrus (BA 18)	
	-22, -78, -8	Left lingual gyrus (BA 18)	
Cluster 3	18, -62, 52 (6.31)	Right superior parietal lobule (BA 7)	32
	22, -56, 56 (5.61)	Right precuneus (BA 7)	
**Faces > Chairs (t-contrast) p > .05, FWE**			
Cluster 1	54, -50, 8 (6.51)	Right superior temporal gyrus (BA 22)	64
**Distinctive > Typical (t-contrast) p > .05, FWE**			
Cluster 1	34, -80, -10 (7.84)	Right fusiform gyrus (BA 19)	293
	44, -80, 0 (7.64)	Right middle occipital gyrus (BA 19)	
	36, -78, -12	Right fusiform gyrus (BA 19)	
	42, -78, -2	Right inferior occipital gyrus (BA 19)	
Cluster 2	-34, -86, -6 (7.15)	Left inferior occipital gyrus (BA 18)	159
	-24, -86, -12 (6.75)	Left inferior occipital gyrus (BA 19)	
	-28, -84, -12	Left fusiform gyrus (BA 19)	
	40, -72, -8 (6.21)	Right inferior occipital gyrus (BA 19)	
	-40, -78, -6	Left inferior occipital gyrus (BA 19)	
	-44, -80, -8	Left inferior occipital gyrus (BA 18)	
**Typical > Distinctive (t-contrast) p > .05, FWE**			
Cluster 1	-4, -64, 24 (6.08)	Left superior parietal lobule (BA 7a)	8
**Predictability (F-contrast) p < .0001, uncorrected**			
Cluster 1	10, 4, 26 (17.79)	Right anterior cingulate gyrus (BA 33)	14
Cluster 2	-10, -2, 30 (17.92)	Left anterior cingulate gyrus (BA 33)	12
**Highly informative cues > uninformative cue (t-contrast) p < .0001, uncorrected**			
		Only two clusters < 3 voxels	
**Interaction between Predictability and Typicality (F-contrast) p < .0001, uncorrected**			
		No significant results	

## 4. Discussion

This study investigated combined effects of stimulus typicality and cue-induced predictability. Our results confirm reports of increased responses to distinctive, as compared to typical stimuli for both face and non-face stimuli. In contrast to our hypotheses, we neither found robust effects of predictability, nor an interaction between predictability and typicality.

### 4.1 The brain is sensitive to visual typicality

We detected stronger brain responses to distinctive, as compared to typical stimuli in all the tested ROIs, which confirms previous findings regarding face typicality encoding in the FFA [28, 52, 53] and extends them to non-face stimuli and their processing in the LOC. Most participants also seem to have noticed variations of typicality either in faces or chairs, when explicitly asked after the experiment. Critically, the lack of interaction with stimulus category indicated that these regions are sensitive to the distance of the stimulus from the prototype regardless of category, which is also implied by our separate analyses of typicality effects for faces and chairs (S2 Fig). Overall, both of these results align with previous findings about the neural effects of typicality (see Section 1.2) and may represent a neural signal encoding distance from the prototype (also see [10], where increasing distance leads to decreasing P200 amplitude). Note that although this idea is more plausible when considering norm-based models [6, 18, 26] it also can be reconciled with exemplar-based models of visual perception [22, 98, 99]. In this case, enhanced brain responses to distinctive stimuli might reflect distances of these from the majority of the others (with no need of abstraction). These responses might then represent a “rarity” signal, based on the fact that distinctive stimuli are also less frequent. Finally, reduced responses to typical stimuli might also represent a phenomenon of neural adaptation to statistical regularities associated with that particular category—when a face is perceived, it is more likely to present certain features—in line with predictive accounts of vision [100]. However, as discussed below, the extent to which such adaptation concerns category-space distances or rather low-level features remains an open question.

We also found increased brain responses to distinctive, as compared to typical stimuli in two large bilateral clusters including the fusiform gyri, the middle and the inferior occipital gyri in our whole-brain analysis. This aligns with the prediction that typicality processing should occur in regions representing and integrating visual features into coherent percepts, such as the LOC [52]. It is interesting to compare our univariate whole-brain results with those of a multivariate study reporting that typical stimuli of two different categories are maximally differentiated in the LOC, in which between-category boundaries are thus maximized [29]. One interpretation may be that the LOC maximally discriminates between categories by storing prototypical representations, such that its responses to prototype-deviant stimuli can be seen as a potential “surprise” signal. We additionally found a small significant cluster of voxels where activity was larger for typical compared to distinctive stimuli, corresponding to the inferior parietal lobule (IPL), which is reminiscent of a cluster identified by [29] in a searchlight analysis. In their study, neural patterns in response to distinctive (atypical) exemplars were more differentiated in the caudal IPL. [29] speculate that this area facilitates recognition of atypical items, achieved by relating these items to the respective category ([29], pp. 175–176). Therefore, stronger activation to typical stimuli within the same area might reflect the activation of prototypical representations necessary to establish the category to which the item belongs. We also note that, while typicality information consolidated through the years (like that to faces and common objects) seems to take place in the visual stream and the IPL, other regions in the frontal lobe and the hippocampus might be involved in learning new prototypes, from entirely new visual categories [24]. The conceptual separation between *structural* and *functional* typicality [1] is thus mirrored at the neural level.

As a cautionary note, the activation differences between typical and distinctive stimuli may reflect differences in low-level features, rather than differences in the location within a hypothetical category space. While it is difficult to determine the extent to which this is the case in the current study and especially in the case of chair images, please note that we equalized luminance and spatial frequency distribution in the case of faces, for which we still found typicality effects in our ROI analysis. Moreover, [52] found no correlation between a neural measure based on physical typicality (height and angle of certain stimulus parts within the same category) and another, based on subjective typicality (ratings). Although we acknowledge that the stimuli used in [52] were more controlled than ours in terms of low-level features and we note that our typicality effects were larger for the less controlled chair stimuli than for faces, these considerations indicate that typicality effects in our study may indeed be driven by category-space structure and not only by low-level features.

### 4.2 Failure to produce cue-induced predictability effects

An important finding is that we detected no main effect of cue-induced predictability in the brain. This likely occurred due to the irrelevance of cue-stimulus contingencies during the task. In fact, while a previous study with a similar cueing design reported lower brain responses in ventral visual areas to strongly expected stimuli [74, 101], also did not find the main effect of cue-based predictability in the fusiform face and parahippocampal place areas. An explanation of the controversial results might be that, in our case and in [74], participants were told explicitly that the cues were task-irrelevant, and this corresponds with subjective reports of low attention to cue contingencies in the present study. By contrast, in [101], participants were explicitly encouraged to learn the cue-stimulus associations and pay attention to them. Moreover, a study where cueing was used for pain conditioning reports activations in the periaqueductal gray when contrasting cues anticipating uncertain pain intensity to those anticipating certain pain intensity [102]. Critically, [102] also instructed participants to pay attention to the cues. Since attention enhances overall cortical responsiveness as well as predictability effects [103, 104], the lack of predictability effects may be a consequence of the lack of such instructions. However, [105] still found predictability effects in ventral visual areas, despite instructing their participants that the contingencies were not task-relevant, similarly to our study and to that of [74]. Another hypothesis is that, as [106] suggest, predictability effects in cueing paradigms depend on the presence of a neutral condition (i.e., when a cue is uninformative about the occurrence of stimulus type). Whereas [105, 107] and [102] only included expected and unexpected conditions, our study and that by [74] also included a neutral condition. Moreover, a recent study using uni- and multi-variate analytic approaches on EEG data from a large sample during a cueing paradigm with contingencies similar to ours found no effects of predictability [75]. Notably, in [75] participants were intensively and successfully trained to learn the cue-stimulus associations, so the lack of effects cannot be ascribed to lack of attention or failure to use the cues. However, even the idea that the effect can only be found in the absence of a neutral condition is debatable, as [101] report predictability effects in face- and object-sensitive regions despite including an uninformative condition similar to ours. Similarly, evidence is mixed even regarding whether participants develop predictions about the single stimulus or, rather, about a stimulus category [106]. In sum, while it seems to be beneficial to ensure active learning and use of cues, and the effect may be larger in the absence of a neutral condition, cueing designs appear to be less robust than previously thought in producing predictability effects. Ideally, a highly-powered, multi-site and multi-method study should systematically assess the impact of task instructions, attention, cue-stimulus contingencies, and category vs. stimulus learning (see further recommendations in [106]).

Another important finding is that, in contrast to what [74] reported, we failed to detect an interaction between predictability and category, the effect on which we based our power analysis. This discrepancy between our results and those of [74], who used nearly identical paradigms and instructions, suggests that this interaction may not necessarily be robust across small differences between the studies (including, for instance, the specific stimulus set used). When inspecting responses for typical and distinctive stimuli separately (Fig 3), we observed a pattern resembling the interaction found by [74], but for typical stimuli only. Although the three-way interaction between category, typicality and predictability was not significant, this pattern suggests that the highly typical faces and buildings used by [74] may have produced enhanced category processing, despite the lower number of participants in their study.

### 4.3 Future investigations on the interaction between typicality and predictability

Critical to our original research question, we detected no interaction between stimulus typicality and predictability, neither in our ROIs, nor at the whole-brain level. We also did not find any three-way interaction between typicality, category, and predictability at the ROI level (see Table 2). Our main explanation for the lack of such interactions is the lack of predictability effects in the present study, discussed in Section 4.2. An alternative possibility is that, in our manipulations, typicality had been formed through years of exposure to stimuli (corresponding to what [1] called “structural typicality”). In contrast, predictability was only induced by verbal instructions, and via prior learning of cue-stimulus associations bearing no semantic values in themselves. Additionally, predictability was not task-relevant. The fact that we found strong effects of typicality–whereas predictability effects were absent in the regions of interest and in the rest of the brain–suggests that the two manipulations did not affect neural processing comparably (also see Section 4.3). Finally, we cannot completely exclude the possibility that typicality simply does not modulate predictability effects. This would imply a theoretical separation between typicality- and predictability-based neural processing advantages. While predictable stimuli invariably produce smaller prediction error signals [108, 109], lower responses to typical stimuli might reflect mechanisms such as neural sharpening [110] or facilitation [111] to commonly seen features.

However, at present these results highlight that cueing designs may not lead to highly robust neural effects, and this affects our ability to make conclusive statements on the presence of interactive vs. additive effects between the two variables, and on their respective theoretical implications. As stated above, further research is needed to develop a robust cueing paradigm. Afterwards, future studies would be able to test whether typicality and predictability rely on similar or related neural mechanisms by using novel stimulus categories (e.g., [24]), by applying another, different predictability-inducing paradigm (see [84] for suggestions) and by comparing different models of neural responses [74]. Such experiments would allow us to i) manipulate predictability and typicality formed at the same time, ii) detect predictability effects that are strong enough to open the possibility for an interaction (e.g., [108, 112]), and iii) establish whether the neural mechanisms driving typicality and predictability are the same.

### 4.4 Category effects

Finally, our ROI analysis revealed clear differences in the effects of stimulus category between the FFA and the LOC, with stronger responses to faces and to chairs in the two respective areas. In addition, we observed activations in object-responsive regions in the ventral visual stream for chairs [93, 94]. By contrast, the whole-brain responses to faces did not reveal any regions of the core face processing network [113, 114]. Instead, faces elicited stronger responses in the rSTG. Typically, studies report face responses in the right inferior/middle temporal gyri (see convergent results in [115] and [114]) and in the superior temporal sulcus (reviewed by [116]), whereas the STG is rarely reported in face research. However, some studies report increased responses in the rSTG to other people’s faces as compared to one’s own [117], and responses to the opposite contrast in its left homotopic area [118, 119]. In our task, participants saw faces that looked like people their age, that they could encounter in real life, so they might have recruited self-other distinction processes more in response to faces, as compared to chairs.

## 5. Conclusions

We found that distinctive stimuli elicited larger responses in face- and object-responsive regions of the ventral visual stream in ROI analyses, and spatially extended effects encompassing these regions when considering whole-brain analyses. The stronger brain responses to distinctive stimuli are in line with the idea that stimuli that are distant from the prototype (or general tendency of a given category) require more neural resources, and thus produce increased signals. These could be interpreted as a signal of distance from the prototype in the context of prototype-based models, or a rarity signal in the context of exemplar-based models. As a caveat, they could also partly represent differences in low-level features between typical and distinctive stimuli. The present typicality effects seem to be independent of cue-induced predictability. However, we failed to produce predictability effects with our cueing paradigm and, importantly, we did not replicate the interaction between predictability and category reported by [74]. Thus, our research question on the interaction between typicality and predictability remains open, and we provide here some directions for future studies.

## Supporting information

S1 Table Summary of post-experimental survey. Synopsis of the post-experimental survey completed by participants Each participant was first asked about whether they experienced any form of discomfort during the procedure, and if so, which type of discomfort. Then they were asked to indicate the degree to which they found themselves paying attention to cue-category contingencies during the scanning session. Their verbal reports (e.g., “never”, not really”, “at the beginning only”, “sometimes”, etc.) were then transferred to a scale ranging from 0 (= “not at all”) to 3 (= “very frequently/always”). No participant reported a score of 3. Participants were then asked whether the faces and the chairs shown during the tasks looked ordinary to them or whether they had something distinctive. Their responses were coded as “yes” if they made observations about differential typicality within that category (e.g., “some of the chairs were quite special”), and with “no” if all items of that category appeared of equal typicality to them (e.g., “all faces looked pretty normal to me, none stood out”). As shown in the total proportions of “yes” responses, typicality manipulations were detected much more often in chairs than faces.
(DOCX)

S2 Table ROI locations in individual participants. MNI coordinates of activation peaks in individual participants during functional localizer run. Right and left fusiform faces areas (FFAs) were localized using the contrast Faces > Objects (or Faces > Noise when the first did not reveal any peak in the approximate area). Right and left lateral occipital complexes (LOCs) were localized using the contrast Objects > Noise. Stars (*) indicate that the coordinate was retrieved when changing the threshold from *p_FWE* < .05 to *p_*uncorrected < .001. In a few cases no peaks were found for a particular area. When comparing this table to S1 Fig. and Fig 3, note that even though we were able to locate these ROIs in nearly all participants, at times the hemodynamic function extracted at the respective location was not of sufficient quality, thus data were excluded from the analysis reported in the main text.
(DOCX)

S3 Table Contrast weights for the whole-brain analysis. Conditions are abbreviated as follows: H = high predictability cue (75% contingencies); M = medium/uninformative cue (50% contingencies); L = low predictability cue (25% contingencies); F = face stimulus; C = chair stimulus; T = typical stimulus; D = distinctive stimulus. Note that weights are set to 0 for target trials and for the six nuisance regressors.
(DOCX)

S1 Fig ANOVA results in individual ROIs. (TIF)

S2 Fig Whole-brain typicality effects for faces and chairs separately. Whole-brain effects of typicality in faces and chairs separately (directional t-contrasts). Results for the contrast distinctive chairs > typical chairs are thresholded at an alpha level of .05 and a family-wise error correction for multiple comparison (FWE, *p* < .05). The other contrasts are thresholded at an alpha level of .001 and no correction (uncorrected, *p* < .001). The ink transparency in the legend visually conveys the different thresholding conservativeness of the results. This figure shows that the effects of distinctiveness (distinctive > typical) tend to occur in overlapping regions for both stimulus types (note that, with an uncorrected threshold, the clusters related to chair distinctiveness overlap with those related to face distinctiveness). Conversely, typicality effects seem to be differently distributed and more prominent for chairs.
(TIF)

S1 File “Raw output of the Anatomy Toolbox”. Labels assigned to the significant activation clusters by means of the Anatomy Toolbox (https://www.fz-juelich.de/en/inm/inm-7/resources/jubrain-anatomy-toolbox). Whole-brain maps resulting from our contrasts of interest were entered in the toolbox. The contrast as well as the coordinates of maximal activation, the size of the activation cluster and the brain regions most likely included in the cluster are reported in the file. This file is provided separately.
(PDF)

S1 Checklist *PLOS ONE* clinical studies checklist. (DOCX)

## References

[1] Dieciuc MA, Folstein JR. Typicality: Stable structures and flexible functions. Psychon Bull Rev [Internet]. 2019;26(2):491–505. Available from: doi: 10.3758/s13423-018-1546-2 30484081

[2] Drucker DM, Aguirre GK. Different Spatial Scales of Shape Similarity Representation in Lateral and Ventral LOC. Cereb Cortex [Internet]. 2009;19(10):2269–80. Available from: doi: 10.1093/cercor/bhn244 19176637 PMC2742590

[3] Edelman S. Representation is representation of similarities. Behav Brain Sci. 1998 Aug;21(4):449–98. doi: 10.1017/s0140525x98001253 10097019

[4] Gaißert N, Wallraven C, Bülthoff HH. Visual and haptic perceptual spaces show high similarity in humans. J Vis [Internet]. 2010;10(11):2. Available from: doi: 10.1167/10.11.2 20884497

[5] Rosch E, Mervis CB. Family resemblances: Studies in the internal structure of categories. Cogn Psychol. 1975;7:573–605.

[6] Valentine T. A unified account of the effects of distinctiveness, inversion, and race in face recognition. Q J Exp Psychol Sect A. 1991;43(2):161–204. doi: 10.1080/14640749108400966 1866456

[7] Valentine T, Bruce V. Recognizing familiar faces: the role of distinctiveness and familiarity. Can J Psychol. 1986 Sep;40(3):300–5. doi: 10.1037/h0080101 3768805

[8] Neumann PG. Visual prototype formation with discontinuous representation of dimensions of variability. Vol. 5, Memory & Cognition. US: Psychonomic Society; 1977. p. 187–97. doi: 10.3758/BF03197361 24202810

[9] Tanaka JW, Kantner J, Bartlett M. How category structure influences the perception of object similarity: The atypicality bias. Front Psychol. 2012;3(JUN):1–11. doi: 10.3389/fpsyg.2012.00147 22685441 PMC3368386

[10] Wuttke SJ, Schweinberger SR. The P200 predominantly reflects distance-to-norm in face space whereas the N250 reflects activation of identity-specific representations of known faces. Biol Psychol [Internet]. 2019;140(November 2018):86–95. Available from: doi: 10.1016/j.biopsycho.2018.11.011 30529289

[11] Valentine T, Lewis MB, Hills PJ. Face-space: A unifying concept in face recognition research. Q J Exp Psychol. 2016;69(10):1996–2019. doi: 10.1080/17470218.2014.990392 25427883

[12] Jenkins R, Dowsett AJ, Burton AM. How many faces do people know? Proc R Soc B Biol Sci. 2018;285(1888). doi: 10.1098/rspb.2018.1319 30305434 PMC6191703

[13] Crouzet SM, Kirchner H, Thorpe SJ. Fast saccades toward faces: Face detection in just 100 ms. J Vis [Internet]. 2010;10(4):16. Available from: doi: 10.1167/10.4.16 20465335

[14] Ro T, Russell C, Lavie N. Changing Faces: A Detection Advantage in the Flicker Paradigm. Psychol Sci [Internet]. 2001 Jan 1;12(1):94–9. Available from: doi: 10.1111/1467-9280.00317 11294237

[15] Carbon CC, Strobach T, Langton SRH, Harsányi G, Leder H, Kovács G. Adaptation effects of highly familiar faces: Immediate and long lasting. Mem Cognit [Internet]. 2007;35(8):1966–76. Available from: doi: 10.3758/BF03192929 18265612

[16] Faerber SJ, Kaufmann JM, Schweinberger SR. Early temporal negativity is sensitive to perceived (rather than physical) facial identity. Neuropsychologia [Internet]. 2015;75:132–42. Available from: http://www.sciencedirect.com/science/article/pii/S0028393215300385 doi: 10.1016/j.neuropsychologia.2015.05.023 26013406

[17] Kloth N, Rhodes G, Schweinberger SR. Watching the brain recalibrate: Neural correlates of renormalization during face adaptation. Neuroimage. 2017;155:1–9. doi: 10.1016/j.neuroimage.2017.04.049 28438667

[18] Rhodes G, Jeffery L. Adaptive norm-based coding of facial identity. Vision Res. 2006;46(18):2977–87. doi: 10.1016/j.visres.2006.03.002 16647736

[19] Faerber SJ, Kaufmann JM, Leder H, Martin EM, Schweinberger SR. The role of familiarity for representations in norm-based face space. PLoS One. 2016;11(5):1–15. doi: 10.1371/journal.pone.0155380 27168323 PMC4864226

[20] Das-Smaal EA, De Swart JH. Variation within categories. Acta Psychol (Amst) [Internet]. 1984;57(3):165–92. Available from: https://www.sciencedirect.com/science/article/pii/0001691884900301

[21] Ross D, Deroche M, Palmeri TJ. Not Just the Norm: Exemplar-Based Models also Predict Face Aftereffects. Psychol Bullettin [Internet]. 2014;21(1):47–70. Available from: https://www.ncbi.nlm.nih.gov/pmc/articles/PMC3624763/pdf/nihms412728.pdf doi: 10.3758/s13423-013-0449-5 23690282 PMC4151123

[22] Lewis M. Face‐space‐R: Towards a unified account of face recognition. Vis cogn [Internet]. 2004 Jan 1;11(1):29–69. Available from: 10.1080/13506280344000194

[23] Giese MA, Leopold DA. Physiologically inspired neural model for the encoding of face spaces. Neurocomputing [Internet]. 2005;65–66:93–101. Available from: https://www.sciencedirect.com/science/article/pii/S0925231204004485

[24] Bowman CR, Iwashita T, Zeithamova D. Tracking prototype and exemplar representations in the brain across learning. Behrens TE, Barense M, Barense M, Tompary A, editors. Elife [Internet]. 2020 Nov;9:e59360. Available from: doi: 10.7554/eLife.59360 33241999 PMC7746231

[25] Panis S, Vangeneugden J, Op de Beeck HP, Wagemans J. The representation of subordinate shape similarity in human occipitotemporal cortex. J Vis. 2008;8(10):1–15. doi: 10.1167/8.10.9 19146351

[26] Panis S, Wagemans J, Op de Beeck HP. Dynamic norm-based encoding for unfamiliar shapes in human visual cortex. J Cogn Neurosci. 2011;23(7):1829–43. doi: 10.1162/jocn.2010.21559 20807059

[27] Wallis G. Toward a unified model of face and object recognition in the human visual system. Front Psychol. 2013;4(AUG):1–25. 23966963 10.3389/fpsyg.2013.00497 PMC3744012

[28] Carlin JD, Kriegeskorte N. Adjudicating between face-coding models with individual-face fMRI responses. PLoS Comput Biol. 2017;13(7):1–28. doi: 10.1371/journal.pcbi.1005604 28746335 PMC5550004

[29] Iordan MC, Greene MR, Beck DM, Fei-Fei L. Typicality sharpens category representations in object-selective cortex. Neuroimage. 2016;134:170–9. doi: 10.1016/j.neuroimage.2016.04.012 27079531 PMC4912889

[30] Mack ML, Preston AR, Love BC. Decoding the Brain’s Algorithm for Categorization from Its Neural Implementation. Curr Biol [Internet]. 2013;23(20):2023–7. Available from: https://www.sciencedirect.com/science/article/pii/S0960982213010415 doi: 10.1016/j.cub.2013.08.035 24094852 PMC3874407

[31] Beck AK, Czernochowski D, Lachmann T, Barahona-Correa B, Carmo JC. Is the dolphin a fish? ERP evidence for the impact of typicality during early visual processing in ultra-rapid semantic categorization in autism spectrum disorder. J Neurodev Disord. 2022;14(1):1–20. 35999495 10.1186/s11689-022-09457-7 PMC9400242

[32] Holmes SJ, Ellis AW. Age of acquisition and typicality effects in three object processing tasks. Vis cogn [Internet]. 2006 May 1;13(7–8):884–910. Available from: 10.1080/13506280544000093

[33] Rosch EH, Simpson C, Miller RS. Structural bases of typicality effects. J Exp Psychol Hum Percept Perform. 1976;2:491–502.

[34] Valentine T, Bruce V. The effects of distinctiveness in recognising and classifying faces. Perception. 1986;15(5):525–35. doi: 10.1068/p150525 3588212

[35] Kayaert G, Op de Beeck HP, Wagemans J. Dynamic prototypicality effects in visual search. J Exp Psychol Gen. 2011;140(3):506–19. doi: 10.1037/a0023494 21668128

[36] Prunty JE, Jenkins R, Qarooni R, Bindemann M. Ingroup and outgroup differences in face detection. Br J Psychol [Internet]. 2023 May 1;114(S1):94–111. Available from: doi: 10.1111/bjop.12588 35876334

[37] Bartlett JC, Hurry S, Thorley W. Typicality and familiarity of faces. Mem \& Cogn. 1984;12:219–28. doi: 10.3758/bf03197669 6472103

[38] Kaufmann JM, Schulz C, Schweinberger SR. High and low performers differ in the use of shape information for face recognition. Neuropsychologia. 2013;51(7):1310–9. doi: 10.1016/j.neuropsychologia.2013.03.015 23562837

[39] Kaufmann J, Vogt S, Schweinberger S. The big nose bias, or when distinctiveness hinders face learning: Evoking an other-race effect with selectively manipulated same-race faces. J Vis. 2018;18(10):1101.

[40] Lee KJ, Perrett D. Presentation-Time Measures of the Effects of Manipulations in Colour Space on Discrimination of Famous Faces. Perception [Internet]. 1997 Jun 1;26(6):733–52. Available from: doi: 10.1068/p260733 9474343

[41] Light LL, Kayra-Stuart F, Hollander S. Recognition memory for typical and unusual faces. J Exp Psychol Hum Learn. 1979 May;5(3):212–28. 528913

[42] Posner MI, Keele SW. On the Genesis of Abstract Ideas. Cognitive psychology: Key readings. New York, NY, US: Psychology Press; 2004. 472–481 p. (Key readings in cognition.). 10.1037/h0025953 5665566

[43] Mauro R, Kubovy M. Caricature and face recognition. Mem Cognit [Internet]. 1992;20(4):433–40. Available from: doi: 10.3758/BF03210927 1495405

[44] Rhodes G. Superportraits: Caricatures and recognition. Psychology Press; 1997.

[45] Wallis G, Siebeck UE, Swann K, Blanz V, Bülthoff HH. The prototype effect revisited: Evidence for an abstract feature model of face recognition. J Vis [Internet]. 2008 Mar 24;8(3):20. Available from: doi: 10.1167/8.3.20 18484826

[46] Schulz C, Kaufmann JM, Walther L, Schweinberger SR. Effects of anticaricaturing vs. caricaturing and their neural correlates elucidate a role of shape for face learning. Neuropsychologia. 2012 Aug;50(10):2426–34. doi: 10.1016/j.neuropsychologia.2012.06.013 22750120

[47] Winograd E. Elaboration and distinctiveness in memory for faces. Vol. 7, Journal of Experimental Psychology: Human Learning and Memory. US: American Psychological Association; 1981. p. 181–90. 7241060

[48] Kaufmann JM, Schweinberger SR. Distortions in the brain? ERP effects of caricaturing familiar and unfamiliar faces. Brain Res. 2008;1228:177–88. doi: 10.1016/j.brainres.2008.06.092 18634766

[49] Itz ML, Golle J, Luttmann S, Schweinberger SR, Kaufmann JM. Dominance of texture over shape in facial identity processing is modulated by individual abilities. Br J Psychol. 2017;108(2):369–96. doi: 10.1111/bjop.12199 27230305

[50] O’Reilly RC, Rudy JW. Conjunctive representations in learning and memory: principles of cortical and hippocampal function. Psychol Rev. 2001 Apr;108(2):311–45. doi: 10.1037/0033-295x.108.2.311 11381832

[51] Davis T, Poldrack RA. Measuring neural representations with fMRI: practices and pitfalls. Ann N Y Acad Sci [Internet]. 2013;1296(1):108–34. Available from: https://nyaspubs.onlinelibrary.wiley.com/doi/abs/10.1111/nyas.12156 23738883 10.1111/nyas.12156

[52] Davis T, Poldrack RA. Quantifying the Internal Structure of Categories Using a Neural Typicality Measure. Cereb Cortex [Internet]. 2013;24(7):1720–37. Available from: doi: 10.1093/cercor/bht014 23442348

[53] Loffler G, Yourganov G, Wilkinson F, Wilson HR. fMRI evidence for the neural representation of faces. Nat Neurosci. 2005;8(10):1386–91. doi: 10.1038/nn1538 16136037

[54] Bestelmeyer PEG, Latinus M, Bruckert L, Rouger J, Crabbe F, Belin P. Implicitly perceived vocal attractiveness modulates prefrontal cortex activity. Cereb Cortex. 2012;22(6):1263–70. doi: 10.1093/cercor/bhr204 21828348

[55] Latinus M, McAleer P, Bestelmeyer PEG, Belin P. Norm-Based Coding of Voice Identity in Human Auditory Cortex. Curr Biol [Internet]. 2013;23(12):1075–80. Available from: https://www.sciencedirect.com/science/article/pii/S096098221300496X doi: 10.1016/j.cub.2013.04.055 23707425 PMC3690478

[56] Halit H, De Haan M, Johnson MH. Modulation of event-related potentials by prototypical and atypical faces. Neuroreport. 2000;11(9):1871–5. doi: 10.1097/00001756-200006260-00014 10884035

[57] Kaufmann JM, Schweinberger SR. The faces you remember: Caricaturing shape facilitates brain processes reflecting the acquisition of new face representations. Biol Psychol. 2012;89(1):21–33. doi: 10.1016/j.biopsycho.2011.08.011 21925235

[58] Schroeger A, Ficco L, Wuttke SJ, Kaufmann JM, Schweinberger SR. Differences between high and low performers in face recognition in electrophysiological correlates of face familiarity and distance-to-norm. Biol Psychol [Internet]. 2023;182(July):108654. Available from: doi: 10.1016/j.biopsycho.2023.108654 37549807

[59] Hauk O, Patterson K, Woollams A, Cooper-Pye E, Pulvermüller F, Rogers TT. How the camel lost its hump: the impact of object typicality on event-related potential signals in object decision. J Cogn Neurosci. 2007 Aug;19(8):1338–53. doi: 10.1162/jocn.2007.19.8.1338 17651007

[60] Ellis AE, Nelson CA. Category prototypicality judgments in adults and children: Behavioral and electrophysiological correlates. Dev Neuropsychol. 1999;15(2):193–211.

[61] Dunn BR, Dunn DA, Languis M, Andrews D. The Relation of ERP Components to Complex Memory Processing. Brain Cogn [Internet]. 1998;36(3):355–76. Available from: https://www.sciencedirect.com/science/article/pii/S0278262698909988 doi: 10.1006/brcg.1998.0998 9647684

[62] Rao RP, Ballard DH. Predictive coding in the visual cortex: a functional interpretation of some extra-classical receptive-field effects. Nat Neurosci. 1999 Jan;2(1):79–87. doi: 10.1038/4580 10195184

[63] Clark A. Whatever next? Predictive brains, situated agents, and the future of cognitive science. Behav Brain Sci [Internet]. 2013;36(3):181–204. Available from: http://www.ncbi.nlm.nih.gov/pubmed/23663408 doi: 10.1017/S0140525X12000477 23663408

[64] Friston K. The free-energy principle: a unified brain theory? Nat Rev Neurosci [Internet]. 2010;11(2):127–38. Available from: http://www.ncbi.nlm.nih.gov/pubmed/20068583 doi: 10.1038/nrn2787 20068583

[65] Friston K, Kiebel S. Predictive coding under the free-energy principle. Philos Trans R Soc B Biol Sci. 2009;364(1521):1211–21. doi: 10.1098/rstb.2008.0300 19528002 PMC2666703

[66] Bastos AM, Usrey WM, Adams RA, Mangun GR, Fries P, Friston KJ. Canonical Microcircuits for Predictive Coding. Neuron [Internet]. 2012;76(4):695–711. Available from: 10.1016/j.neuron.2012.10.038 23177956 PMC3777738

[67] Keller GB, Mrsic-Flogel TD. Predictive Processing: A Canonical Cortical Computation. Neuron [Internet]. 2018;100(2):424–35. Available from: doi: 10.1016/j.neuron.2018.10.003 30359606 PMC6400266

[68] D’Astolfo L, Rief W. Learning about expectation violation from prediction error paradigms—A meta-analysis on brain processes following a prediction error. Front Psychol. 2017;8(JUL):1–11. 28804467 10.3389/fpsyg.2017.01253 PMC5532445

[69] Hohwy J. Prediction error minimization in the brain. Routledge Handb Comput mind. 2018;159–72.

[70] Friston K. The history of the future of the Bayesian brain. Neuroimage [Internet]. 2012;62(2):1230–3. Available from: https://www.sciencedirect.com/science/article/pii/S1053811911011657 doi: 10.1016/j.neuroimage.2011.10.004 22023743 PMC3480649

[71] Knill DC, Pouget A. The Bayesian brain: the role of uncertainty in neural coding and computation. Trends Neurosci [Internet]. 2004;27(12):712–9. Available from: https://www.sciencedirect.com/science/article/pii/S0166223604003352 doi: 10.1016/j.tins.2004.10.007 15541511

[72] Ganel T, Gonzalez CLR, Valyear KF, Culham JC, Goodale MA, Köhler S. The relationship between fMRI adaptation and repetition priming. Neuroimage [Internet]. 2006;32(3):1432–40. Available from: https://www.sciencedirect.com/science/article/pii/S1053811906006318 doi: 10.1016/j.neuroimage.2006.05.039 16854597

[73] S. S. Separate modifiability, mental modules, and the use of pure and composite measures to reveal them. Acta Psychol (Amst) [Internet]. 2001;106(1–2):147–246. Available from: http://www.embase.com/search/results?subaction=viewrecord&from=export&id=L33454553 11256336 10.1016/s0001-6918(00)00045-7

[74] Egner T, Monti JM, Summerfield C. Expectation and surprise determine neural population responses in the ventral visual stream. J Neurosci. 2010;30(49):16601–8. doi: 10.1523/JNEUROSCI.2770-10.2010 21147999 PMC3975573

[75] den Ouden C, Zhou A, Mepani V, Kovács G, Vogels R, Feuerriegel D. Stimulus expectations do not modulate visual event-related potentials in probabilistic cueing designs. Neuroimage. 2023;280(April). 10.1016/j.neuroimage.2023.120347 37648120

[76] Lakens D, Caldwell AR. Simulation-Based Power Analysis for Factorial Analysis of Variance Designs. Adv Methods Pract Psychol Sci [Internet]. 2021 Jan 1;4(1):2515245920951503. Available from: 10.1177/2515245920951503

[77] Dennett H, Edwards M, McKone E. Are objects like faces? Norm-based versus exemplar-based coding as revealed by adaptation aftereffects. J Vis [Internet]. 2009 Aug 1;9(8):518. Available from: 10.1167/9.8.518

[78] Brainard DH. The Psychophysics Toolbox. Spat Vis. 1997;10(4):433–6. 9176952

[79] Kleiner M, Brainard DH, Pelli D. What’s new in Psychtoolbox-3? Perception. 2007;36:1–16.

[80] Itz ML, Schweinberger SR, Schulz C, Kaufmann JM. Neural correlates of facilitations in face learning by selective caricaturing of facial shape or reflectance. Neuroimage [Internet]. 2014;102:736–47. Available from: http://www.sciencedirect.com/science/article/pii/S1053811914007162 doi: 10.1016/j.neuroimage.2014.08.042 25173417

[81] Limbach K, Itz ML, Schweinberger SR, Jentsch AD, Romanova L, Kaufmann JM. Neurocognitive effects of a training program for poor face recognizers using shape and texture caricatures: A pilot investigation. Neuropsychologia. 2022;165:1–16. doi: 10.1016/j.neuropsychologia.2021.108133 34971671

[82] Zhou X, Itz ML, Vogt S, Kaufmann JM, Schweinberger SR, Mondloch CJ. Similar use of shape and texture cues for own- and other-race faces during face learning and recognition. Vision Res [Internet]. 2021;188(September 2019):32–41. Available from: doi: 10.1016/j.visres.2021.06.014 34280815

[83] Müller-Bardorff M, Schulz C, Peterburs J, Bruchmann M, Mothes-Lasch M, Miltner W, et al. Effects of emotional intensity under perceptual load: An event-related potentials (ERPs) study. Biol Psychol. 2016 May;117:141–9. doi: 10.1016/j.biopsycho.2016.03.006 26995785

[84] Feuerriegel D. Selecting appropriate designs and comparison conditions in repetition paradigms. Cortex [Internet]. 2016;80:196–205. Available from: http://www.sciencedirect.com/science/article/pii/S0010945215003743 doi: 10.1016/j.cortex.2015.10.022 26654854

[85] Cziraki C, Greenlee MW, Kovács G. Neural Correlates of High-Level Adaptation-Related Aftereffects. J Neurophysiol [Internet]. 2010 Jan 13;103(3):1410–7. Available from: doi: 10.1152/jn.00582.2009 20071633

[86] Eickhoff SB, Stephan KE, Mohlberg H, Grefkes C, Fink GR, Amunts K, et al. A new SPM toolbox for combining probabilistic cytoarchitectonic maps and functional imaging data. Neuroimage [Internet]. 2005;25(4):1325–35. Available from: https://www.sciencedirect.com/science/article/pii/S105381190400792X doi: 10.1016/j.neuroimage.2004.12.034 15850749

[87] Lancaster JL, Woldorff MG, Parsons LM, Liotti M, Freitas CS, Rainey L, et al. Automated Talairach Atlas labels for functional brain mapping. Hum Brain Mapp. 2000;10(3):120–31. doi: 10.1002/1097-0193(200007)10:3<120::aid-hbm30>3.0.co;2-8 10912591 PMC6871915

[88] Lancaster JL, Rainey LH, Summerlin JL, Freitas CS, Fox PT, Evans AC, et al. Automated labeling of the human brain: A preliminary report on the development and evaluation of a forward-transform method. Hum Brain Mapp. 1997;5(4):238–42. doi: 10.1002/(SICI)1097-0193(1997)5:4<238::AID-HBM6>3.0.CO;2-4 20408222 PMC2860189

[89] Caspers J, Zilles K, Eickhoff SB, Schleicher A, Mohlberg H, Amunts K. Cytoarchitectonical analysis and probabilistic mapping of two extrastriate areas of the human posterior fusiform gyrus. Brain Struct Funct [Internet]. 2013;218(2):511–26. Available from: doi: 10.1007/s00429-012-0411-8 22488096 PMC3580145

[90] Kanwisher N, McDermott J, Chun MM. The Fusiform Face Area: A Module in Human Extrastriate Cortex Specialized for Face Perception. J Neurosci [Internet]. 1997 Jun 1;17(11):4302 LP– 4311. Available from: http://www.jneurosci.org/content/17/11/4302.abstract doi: 10.1523/JNEUROSCI.17-11-04302.1997 9151747 PMC6573547

[91] Grill-Spector K, Kushnir T, Hendler T, Edelman S, Itzchak Y, Malach R. A sequence of object-processing stages revealed by fMRI in the human occipital lobe. Hum Brain Mapp. 1998;6(4):316–28. doi: 10.1002/(SICI)1097-0193(1998)6:4<316::AID-HBM9>3.0.CO;2-6 9704268 PMC6873387

[92] Malikovic A, Amunts K, Schleicher A, Mohlberg H, Kujovic M, Palomero-Gallagher N, et al. Cytoarchitecture of the human lateral occipital cortex: mapping of two extrastriate areas hOc4la and hOc4lp. Brain Struct Funct [Internet]. 2016;221(4):1877–97. Available from: doi: 10.1007/s00429-015-1009-8 25687261

[93] Grill-Spector K, Kourtzi Z, Kanwisher N. The lateral occipital complex and its role in object recognition. Vision Res [Internet]. 2001;41(10):1409–22. Available from: https://www.sciencedirect.com/science/article/pii/S0042698901000736 doi: 10.1016/s0042-6989(01)00073-6 11322983

[94] Malach R, Reppas JB, Benson RR, Kwong KK, Jiang H, Kennedy WA, et al. Object-related activity revealed by functional magnetic resonance imaging in human occipital cortex. Proc Natl Acad Sci. 1995;92(18):8135–9. doi: 10.1073/pnas.92.18.8135 7667258 PMC41110

[95] Ollinger JM, Shulman GL, Corbetta M. Separating Processes within a Trial in Event-Related Functional MRI: I. The Method. Neuroimage [Internet]. 2001;13(1):210–7. Available from: https://www.sciencedirect.com/science/article/pii/S1053811900907109 doi: 10.1006/nimg.2000.0710 11133323

[96] Brett M, Anton JL, Valabregue R, Poline JB. Region of interest analysis using an SPM toolbox. In: 8th international conference on functional mapping of the human brain. Sendai; 2002. p. 497.

[97] van Doorn J, van den Bergh D, Böhm U, Dablander F, Derks K, Draws T, et al. The JASP guidelines for conducting and reporting a Bayesian analysis. Psychon Bull Rev. 2021 Jun;28(3):813–26. doi: 10.3758/s13423-020-01798-5 33037582 PMC8219590

[98] Ashby FG, Maddox WT. Relations between prototype, exemplar, and decision bound models of categorization. J Math Psychol. 1993;37(3):372–400.

[99] Sigala N, Gabbiani F, Logothetis NK. Visual Categorization and Object Representation in Monkeys and Humans. J Cogn Neurosci [Internet]. 2002 Feb 15;14(2):187–98. Available from: doi: 10.1162/089892902317236830 11970785

[100] Grotheer M, Kovacs GZ. Can predictive coding explain repetition suppression? Cortex [Internet]. 2016;80(Review):113–24. Available from: https://www.sciencedirect.com/science/article/pii/S0010945216000149 doi: 10.1016/j.cortex.2015.11.027 26861559

[101] Dunovan K, Wheeler ME. Computational and neural signatures of pre and post-sensory expectation bias in inferior temporal cortex. Sci Rep [Internet]. 2018;8(1):13256. Available from: doi: 10.1038/s41598-018-31678-x 30185928 PMC6125426

[102] Lin CS, Hsieh JC, Yeh TC, Niddam DM. Predictability-mediated pain modulation in context of multiple cues: An event-related fMRI study. Neuropsychologia [Internet]. 2014;64:85–91. Available from: https://www.sciencedirect.com/science/article/pii/S0028393214003261 doi: 10.1016/j.neuropsychologia.2014.09.021 25258246

[103] Kok P, Rahnev D, Jehee JFM, Lau HC, De Lange FP. Attention reverses the effect of prediction in silencing sensory signals. Cereb Cortex. 2012;22(9):2197–206. doi: 10.1093/cercor/bhr310 22047964

[104] Hsu YF, Hamalainen J, Waszak F. Both attention and prediction are necessary for adaptive neuronal tuning in sensory processing. Front Hum Neurosci. 2014;8:152. doi: 10.3389/fnhum.2014.00152 24723871 PMC3972470

[105] Richter D, de Lange FP. Statistical learning attenuates visual activity only for attended stimuli. Frank MJ, Kahnt T, Wyart V, Heinzle J, editors. Elife [Internet]. 2019;8:e47869. Available from: 10.7554/eLife.47869 PMC6731093 31442202

[106] Feuerriegel D, Vogels R, Kovács G. Evaluating the evidence for expectation suppression in the visual system. Neurosci Biobehav Rev. 2021;126(February):368–81. doi: 10.1016/j.neubiorev.2021.04.002 33836212

[107] Davis B, Hasson U. Predictability of what or where reduces brain activity, but a bottleneck occurs when both are predictable ☆. Neuroimage [Internet]. 2018;167(May 2016):224–36. Available from: 10.1016/j.neuroimage.2016.06.001 27263508

[108] Summerfield C, Monti JM, Trittschuh EH, Mesulam M, Egner T. Neural repetition suppression reflects fulfilled perceptual expectations. Nat Neurosci [Internet]. 2008;11(9):1004–6. Available from: http://www.ncbi.nlm.nih.gov/pubmed/21138698 doi: 10.1038/nn.2163 19160497 PMC2747248

[109] Robinson JE, Woods W, Leung S, Kaufman J, Breakspear M, Young AW, et al. Prediction-error signals to violated expectations about person identity and head orientation are doubly-dissociated across dorsal and ventral visual stream regions. Neuroimage [Internet]. 2020;206(November 2019):116325. Available from: doi: 10.1016/j.neuroimage.2019.116325 31682984

[110] Desimone R. Neural mechanisms for visual memory and their role in attention. Proc Natl Acad Sci U S A. 1996 Nov;93(24):13494–9. doi: 10.1073/pnas.93.24.13494 8942962 PMC33636

[111] Henson RNA, Rugg MD. Neural response suppression, haemodynamic repetition effects, and behavioural priming. Neuropsychologia [Internet]. 2003;41(3):263–70. Available from: https://www.sciencedirect.com/science/article/pii/S0028393202001598 doi: 10.1016/s0028-3932(02)00159-8 12457752

[112] Kovács G, Iffland L, Vidnyánszky Z, Greenlee MW. Stimulus repetition probability effects on repetition suppression are position invariant for faces. Neuroimage. 2012;60(4):2128–35. doi: 10.1016/j.neuroimage.2012.02.038 22387172

[113] Haxby J V, Hoffman EA, Gobbini MI. The distributed human neural system for face perception. Trends Cogn Sci [Internet]. 2000;4(6):223–33. Available from: http://www.sciencedirect.com/science/article/pii/S1364661300014820 doi: 10.1016/s1364-6613(00)01482-0 10827445

[114] Müller VI, Höhner Y, Eickhoff SB. Influence of task instructions and stimuli on the neural network of face processing: An ALE meta-analysis. Cortex. 2018;103:240–55. doi: 10.1016/j.cortex.2018.03.011 29665467 PMC5988961

[115] Fusar-Poli P, Placentino A, Carletti F, Landi P, Allen P, Surguladze S, et al. Functional atlas of emotional faces processing: A voxel-based meta-analysis of 105 functional magnetic resonance imaging studies. J Psychiatry Neurosci. 2009;34(6):418–32. 19949718 PMC2783433

[116] Iidaka T. Role of the fusiform gyrus and superior temporal sulcus in face perception and recognition: An empirical review. Jpn Psychol Res [Internet]. 2014 Jan 1;56(1):33–45. Available from: 10.1111/jpr.12018

[117] Devue C, Collette F, Balteau E, Degueldre C, Luxen A, Maquet P, et al. Here I am: The cortical correlates of visual self-recognition. Vol. 1143, Brain Research. Devue, Christel: Department of Cognitive Science, University of Liege, 5, Boulevard du Rectorat (Bat. B32), Liege, Belgium, 4000, cdevue@ulg.ac.be: Elsevier Science; 2007. p. 169–82. doi: 10.1016/j.brainres.2007.01.055 17306235

[118] Kircher TTJ, Senior C, Phillips ML, Rabe-Hesketh S, Benson PJ, Bullmore ET, et al. Recognizing one’s own face. Cognition [Internet]. 2001;78(1):B1–15. Available from: https://www.sciencedirect.com/science/article/pii/S0010027700001049 doi: 10.1016/s0010-0277(00)00104-9 11062324

[119] Uddin LQ, Kaplan JT, Molnar-Szakacs I, Zaidel E, Iacoboni M. Self-face recognition activates a frontoparietal “mirror” network in the right hemisphere: an event-related fMRI study. Neuroimage [Internet]. 2005;25(3):926–35. Available from: https://www.sciencedirect.com/science/article/pii/S1053811904007669 doi: 10.1016/j.neuroimage.2004.12.018 15808992

