# Supplementary material for: Investigating the neural effects of typicality and predictability for face and object stimuli
Source: PLoS One. 2024 May 22;19(5):e0293781. doi: 10.1371/journal.pone.0293781 (PMC11111078; doi:10.1371/journal.pone.0293781)
Supplement: S1 Table — Synopsis of the post-experimental survey completed by participants Each participant was first asked about whether they experienced any form of discomfort during the procedure, and if so, which type of discomfort. Then they were asked to indicate the degree to which they found themselves paying attention to cue-category contingencies during the scanning session. Their verbal reports (e.g., “never”, not really”, “at the beginning only”, “sometimes”, etc.) were then transferred to a scale ranging from 0 (= “not at all”) to 3 (= “very frequently/always”). No participant reported a score of 3. Participants were then asked whether the faces and the chairs shown during the tasks looked ordinary to them or whether they had something distinctive. Their responses were coded as “yes” if they made observations about differential typicality within that category (e.g., “some of the chairs were quite special”), and with “no” if all items of that category appeared of equal typicality to them (e.g., “all faces looked pretty normal to me, none stood out”). As shown in the total proportions of “yes” responses, typicality manipulations were detected much more often in chairs than faces. (DOCX) [file pone.0293781.s001.docx]

S1 Table. **Summary of post-experimental survey**

Table S1: Synopsis of the post-experimental survey completed by participants Each participant was first asked about whether they experienced any form of discomfort during the procedure, and eventually which ones. Then they were asked to indicate to which degree they found themselves paying attention to cue-category contingencies during the scanning session. Their verbal reports (e.g., “never”, not really”, “at the beginning only”, “sometimes”, etc.) were then transferred to a scale ranging from 0 (= “not at all”) to 3 (= “very frequently/always”). No participant reported a score of 3. Participants were then asked whether the faces and the chairs shown during the tasks look ordinary to them or whether they had something distinctive. Their responses were coded as “yes” if they made observations about differential typicality within that category (e.g., “some of the chairs were quite special”), and with “no” if all items of that category appeared of equal typicality to them (e.g., “all faces looked pretty normal to me, none stood out”). As shown in the total proportions of “yes” responses, typicality manipulations were detected much more often in chairs than faces.

| **SubjectID** | **Discomforts during scanning?** | **Which discomforts?** | **Attention to cue-stimulus contingencies** | **Typicality manipulation in objects?** | **Typicality manipulation in faces?** |
| --- | --- | --- | --- | --- | --- |
| NiKr92 | Yes | itching, remaining still | 1 | Yes | No |
| JoMe03 | Yes | earplugs, head adjustment | 1 | No | No |
| ZiHe98 | Yes | glasses | 1 | Yes | No |
| JoBi01 | No | none | 0 | No | No |
| KeSi01 | No | none | 0 | No | Yes |
| RiJa02 | Yes | head pain | 1 | No | No |
| FeBa02 | No | none | 1 | Yes | No |
| AmKa01 | Yes | staying still, boredom | 2 | Yes | No |
| MoZe01 | Yes | hard to stay focussed | 1 | No | No |
| UlKr94 | Yes | numb fingers | 0 | No | No |
| MiWe97 | Yes | tired | 2 | No | No |
| KaSo01 | Yes | tired, boredom | 1 | No | No |
| PiEl00 | Yes | eye strain right | 2 | No | No |
| PaPo97 | Yes | head pain | 0 | Yes | No |
| KlBeHe01 | No | none | 0 | No | No |
| RoTe91 | No | none | 2 | Yes | No |
| MeBo00 | Yes | tired | 2 | Yes | No |
| LeFi01 | No | none | 0 | Yes | No |
| SiLa01 | No | none | 2 | Yes | Yes |
| PaAlHa96 | Yes | foot pain, tired | 0 | Yes | No |
| ViKl03 | No | none | 2 | No | No |
| **SubjectID** | **Discomforts during scanning?** | **Which discomforts?** | **Attention to cue-stimulus contingencies** | **Typicality manipulation in objects?** | **Typicality manipulation in faces?** |
| LiHo94 | Yes | vibration, tired | 0 | Yes | Yes |
| LaSp91 | Yes | head pain | 0 | Yes | No |
| SeHu99 | Yes | tired | 2 | Yes | No |
| ViIv95 | Yes | tired | 0 | Yes | No |
| RiSi95 | No | none | 1 | Yes | No |
| AmAi98 | Yes | head pain, eye strain | 0 | Yes | No |
| ChRz87 | Yes | head pain | 0 | No | No |
| AnFi01 | No | none | 0 | No | No |
| IlCh95 | Yes | tired, cold | 0 | Yes | No |
| NaSc91 | Yes | earplugs uncomfortable | 0 | Yes | No |
| ReRu02 | No | none | 2 | Yes | No |
| YaEi97 | No | none | 0 | No | No |
| ViPa00 | Yes | boredom, head pain | 1 | Yes | Yes |
| JoKn87 | Yes | cold, tingling, tired | 0 | No | Yes |
| **Summary** | **22/35 “yes”** | **-** | **M = 0,77 (SD = 0,84)** | **20/35 “yes”** | **5/35 “yes”** |
