# Supplementary material for: Investigating the neural effects of typicality and predictability for face and object stimuli
Source: PLoS One. 2024 May 22;19(5):e0293781. doi: 10.1371/journal.pone.0293781 (PMC11111078; doi:10.1371/journal.pone.0293781)
Supplement: S2 Table — MNI coordinates of activation peaks in individual participants during functional localizer run. Right and left fusiform faces areas (FFAs) were localized using the contrast Faces > Objects (or Faces > Noise when the first did not reveal any peak in the approximate area). Right and left lateral occipital complexes (LOCs) were localized using the contrast Objects > Noise. Stars (*) indicate that the coordinate was retrieved when changing the threshold from p_FWE < .05 to p_uncorrected < .001. In a few cases no peaks were found for a particular area. When comparing this table to S1 Fig. and Fig 3, note that even though we were able to locate these ROIs in nearly all participants, at times the hemodynamic function extracted at the respective location was not of sufficient quality, thus data were excluded from the analysis reported in the main text. (DOCX) [file pone.0293781.s002.docx]

S2 Table. **ROI locations in individual participants**

Table S2: MNI coordinates of activation peaks in individual participants during functional localizer block. Right and left fusiform faces areas (FFAs) were localized using the contrast Faces > Objects (or Faces > Noise when the first did not reveal any peak in the approximate area). Right and left lateral occipital complexes (LOCs) were localized using the contrast Objects > Noise. Stars (*) indicate that the coordinate was retrieved when changing the threshold from $p_{FWE}<.05$ to $p_{uncorrected}<.001$. In a few cases no peaks were found for a particular area. When comparing this table to Fig. S1 and Fig. 3, note that even though we were able to locate these ROIs in nearly all participants, at times the hemodynamic function extracted at the respective location was not of sufficient quality, thus data were excluded from the analysis reported in the main text.

| **SubjectID** | **Right FFA** | **Left FFA** | **Right LOC** | **Left LOC** |
| --- | --- | --- | --- | --- |
| NiKr92 | 42, -58, -14 | -44, -52, -22* | 28,-86, -16 | -36, -80, 8 |
| JoMe03 | Not found | -38, -48, -18* | 40, -78, -2 | -50, -72, 0 |
| ZiHe98 | 42, -64, -14* | -44, -62, -16* | 40, -86, 2 | -36, -74, -2 |
| JoBi01 | 48, -58, -29* | -46, -50, -20* | 44, -78, 2 | -38, -90, 8 |
| KeSi01 | 36, -46, -20 | -32, -56, -18 | 38, -72, 0 | -46, -80, 14 |
| RiJa02 | 34, -50, -20* | Not found | 42, -84, -8* | -52, -70, -12 |
| FeBa02 | 40, -60, -14 | -42, -66, -16 | 38, -78, -2 | -48, -82, 0 |
| AmKa01 | 36, -60, -16 | -38, 56, -18 | 38, -92, 4 | -42, -78, 8 |
| MoZe01 | 42, -58, -20 | -44, -62, -18* | 34, -78, 4 | -42, -76, -10 |
| UlKr94 | 42, -42, -18* | -42, -48, -20 | 42, -86, 2 | -36, -92, -2 |
| MiWe97 | 34, -64, -14* | -38, -48, -22* | 46, -72, -2 | -44, -84, -6 |
| KaSo01 | 44, -46, -16 | -44, -46, -18 | 48, -74, 4 | -40, -74, 0 |
| PiEl00 | 40, -54, -20 | -40, -60, -22 | 40, -76, 2 | -50, -76, -8 |
| PaPo97 | 38, -56, -16* | -32, -56, -16 | 38, -82, -8 | -36, -82, -2 |
| KlBeHe01 | 44, -46, -20* | -40, -52, -20* | 44, -66, -6 | -52, -70, -2 |
| RoTe91 | 42, -64, -14 | -42, -52, -22 | 38, -82, -4 | -46, -76, -6 |
| MeBo00 | 36, -48, -20 | -38, -46, -18 | 32, -94, 8 | -38, -90, 4 |
| LeFi01 | 38, -48, -22* | -38, -56, -20 | 40, -78, -4 | -48, -72, 0 |
| SiLa01 | 40, -52, -18* | -38, -52, -20* | 46, -80, -4 | -42, 72, -12 |
| PaAlHa96 | 46, -64, -20 | -38, -46, -22 | 50, -78, -4 | -50, -70, -4 |
| ViKl03 | 46, -52, -16 | -38 -56, -16* | 52, -70, -8 | -34, -84, 0 |
| LiHo94 | 36, -48, -20 | -34, -48, -24 | 50, -70, 2 | -48, -82, -6 |
| LaSp91 | 36, -40, -24* | -38, -42, -16* | 40, -86, 2 | -48, -80, -10 |
| SeHu99 | 42, -44, -14 | -36, -66, -4 | 46, -78, -14 | -48, -78, 0 |
| ViIv95 | 54, -66, -14 | Not found | 50, -80, 6 | -48, -82, -4 |
| **SubjectID** | **Right FFA** | **Left FFA** | **Right LOC** | **Left LOC** |
| RiSi95 | 44, -58, -22 | -38, -40, -26 | 46, -80, 6 | -4, -80, 2 |
| AmAi98 | 48, -54, -24* | -40, -48, -20* | 44, -84, -14 | -42, -86, -8 |
| ChRz87 | 48, -62, -18 | -42, -58, -16* | 40, -70, -6* | -38, -72, -10* |
| AnFi01 | 38, -56, -16 | -36, -62, -16 | 48, -86, 2 | -46, -76, -2 |
| IlCh95 | 36, -48, -14 | -38, -54, -18 | 46, -80, -4 | -48, -78, -6 |
| NaSc91 | 38, -46, -24* | -38, -44, -16* | 44, -80, -4 | -40, -72, -12 |
| ReRu02 | 36, -52, -16 | -32, -58, -20* | 42, -86, 4 | -46, -78, 0 |
| YaEi97 | 38, -46, -18 | -38, -48, -24 | 32, -88, 6 | -46, -80, -4 |
| ViPa00 | 40, -42, -26 | -40, -44, -26 | 40, -82, -2 | -42, -76, -8 |
| JoKn87 | 38, -5, -18* | -38, -52, -18 | 42, -78, -1 | -44, -78, -4 |
| **Average(SD)** | **x = 40,6(4,7)**  **y = -52,9(7,7)**  **z = -18,6(3,9)** | **x = -38,9(3,5)**  **y = -52,4(6,9)**  **z = -18,9(4,0)** | **x = 40,1(4,6)**  **y = -76,1(8,2)**  **z = -3,6(6,4)** | **x = -43,5(5,1)**  **y = -78,3(6,6)**  **z = -3,5(5,7)** |
