# Supplementary material for: Investigating the neural effects of typicality and predictability for face and object stimuli
Source: PLoS One. 2024 May 22;19(5):e0293781. doi: 10.1371/journal.pone.0293781 (PMC11111078; doi:10.1371/journal.pone.0293781)
Supplement: S3 Table — Conditions are abbreviated as follows: H = high predictability cue (75% contingencies); M = medium/uninformative cue (50% contingencies); L = low predictability cue (25% contingencies); F = face stimulus; C = chair stimulus; T = typical stimulus; D = distinctive stimulus. Note that weights are set to 0 for target trials and for the six nuisance regressors. (DOCX) [file pone.0293781.s003.docx]

S3 Table. Contrast weights for the whole-brain analysis

*Table S3: Conditions are abbreviated as follows: H = high predictability cue (75% contingencies); M = medium/uninformative cue (50% contingencies); L = low predictability cue (25 % contingencies); F = face stimulus; C = chair stimulus; T = typical stimulus; D = distinctive stimulus. Note that weights are set to 0 for target trials and for the six nuisance regressors.*

| **Contrast weights for the whole brain analysis**  **Conditions**   \| H_F_T \| H_F_D \| H_C_T \| H_C_D \| M_F_T \| M_F_D \| M_C_T \| M_C_D \| L_F_T \| L_F_D \| L_C_T \| L_C_D \| Target_trials and  six nuisance regressors \| \| --- \| --- \| --- \| --- \| --- \| --- \| --- \| --- \| --- \| --- \| --- \| --- \| --- \| | |
| --- | --- | --- | --- | --- | --- | --- | --- | --- | --- | --- | --- | --- | --- | --- |
| Main effects of category | t-contrast: Faces > Chairs [1 1 -1 -1 1 1 -1 -1 1 1 -1 -1 0 0 0 0 0 0 0]  t-contrast: Chairs > Faces [-1 -1 1 1 -1 -1 1 1 -1 -1 1 1 0 0 0 0 0 0 0] |
| Main effects of typicality | t-contrast: Distinctive > Typical [-1 1 -1 1 -1 1 -1 1 -1 1 -1 1 0 0 0 0 0 0 0]  t-contrast: Typical > Distinctive [1 -1 1 -1 1 -1 1 -1 1 -1 1 -1 0 0 0 0 0 0 0] |
| Main effects of predictability | Directional effect (t-contrast: High Face Predictability and High Chair Predictability > Uninformative Cue)  [1 1 1 1 -2 -2 -2 -2 1 1 1 1 0 0 0 0 0 0 0]  Non-directional effect (f-contrast: [1 1 1 1 -1 -1 -1 -1 0 0 0 0 0 0 0 0 0 0 0  0 0 0 0 -1 -1 -1 -1 1 1 1 1 0 0 0 0 0 0 0]) |
| Interaction between typicality and predictability | f-contrast: [1 -1 1 -1 -1 1 -1 1 0 0 0 0 0 0 0 0 0 0 0  0 0 0 0 1 -1 1 -1 -1 1 -1 1 0 0 0 0 0 0 0] |
