# Supplementary figures and images for: Investigating the neural effects of typicality and predictability for face and object stimuli
Source: PLoS One. 2024 May 22;19(5):e0293781. doi: 10.1371/journal.pone.0293781 (PMC11111078; doi:10.1371/journal.pone.0293781)

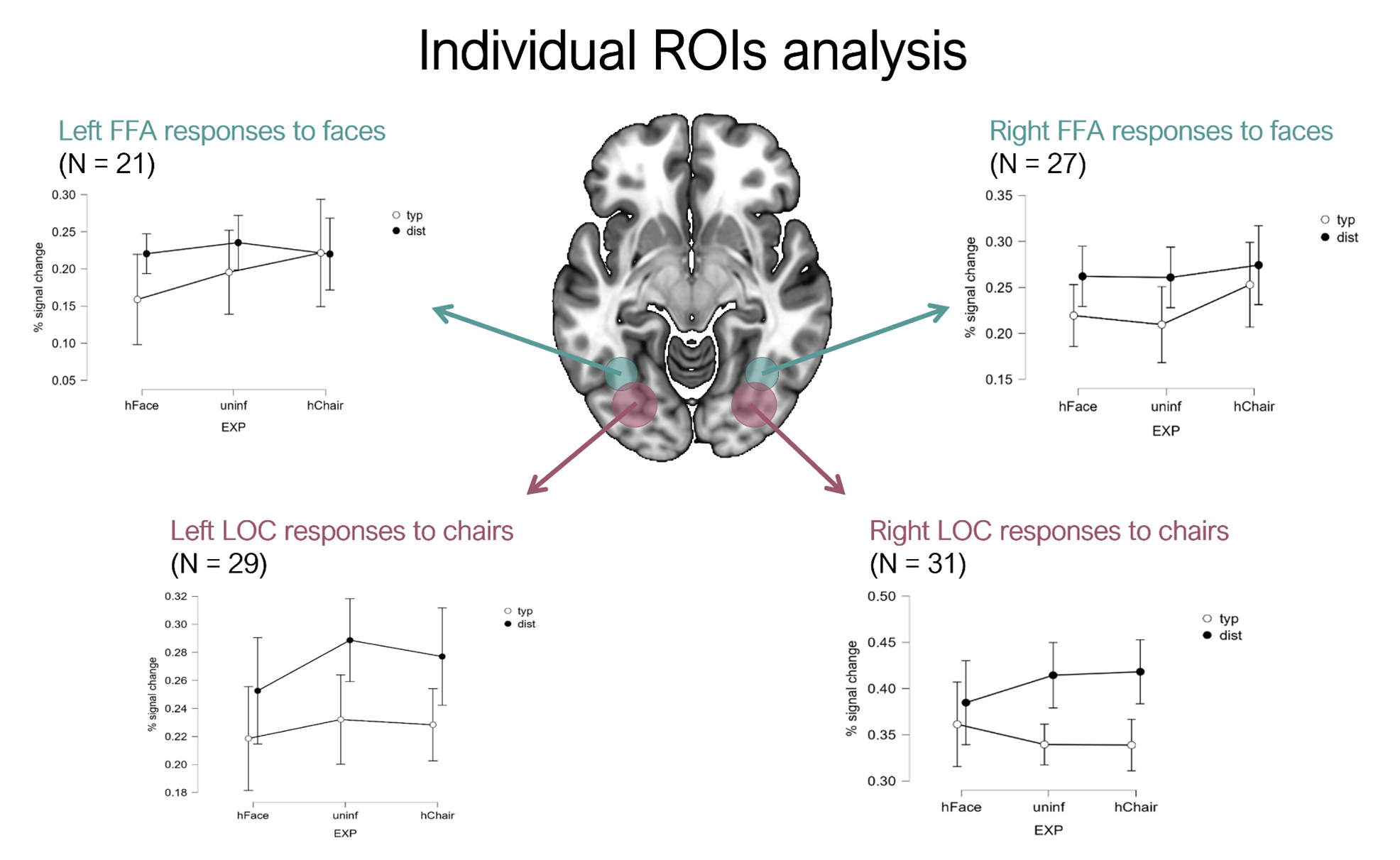

Supplement: S1 Fig — (TIF) [file pone.0293781.s004.tif]

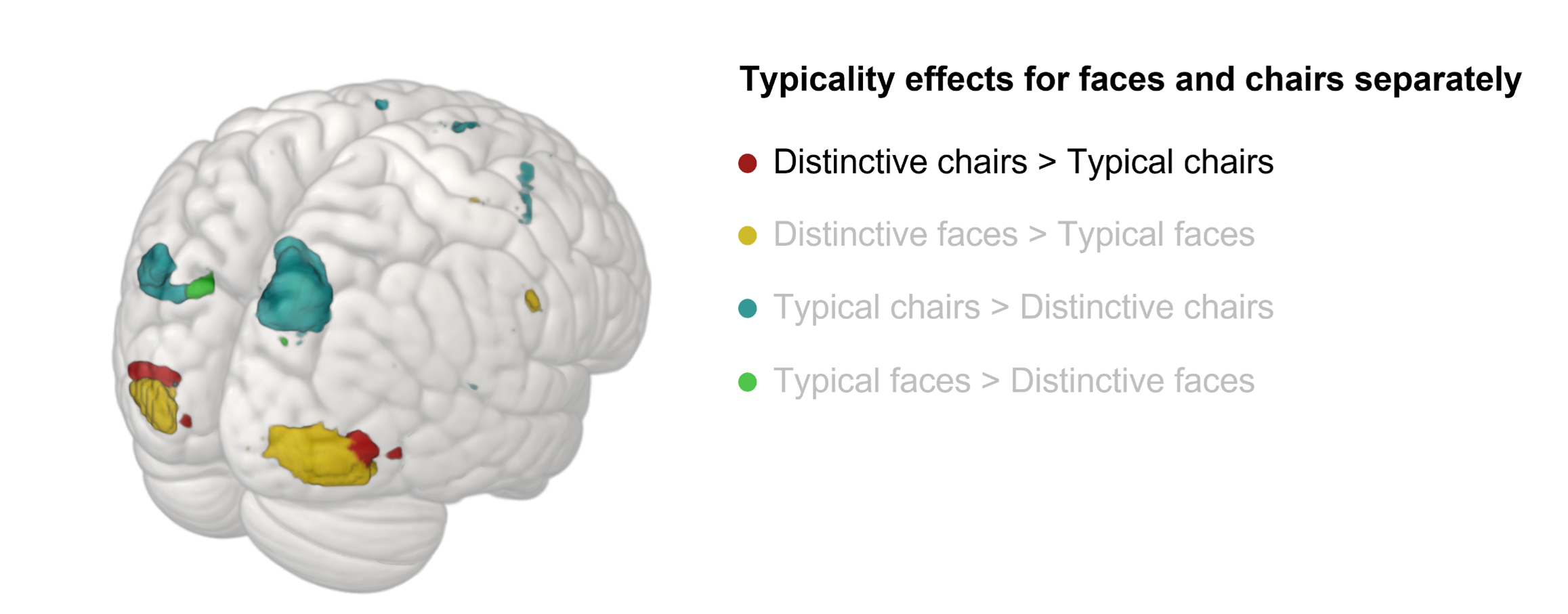

Supplement: S2 Fig — Whole-brain effects of typicality in faces and chairs separately (directional t-contrasts). Results for the contrast distinctive chairs > typical chairs are thresholded at an alpha level of .05 and a family-wise error correction for multiple comparison (FWE, p < .05). The other contrasts are thresholded at an alpha level of .001 and no correction (uncorrected, p < .001). The ink transparency in the legend visually conveys the different thresholding conservativeness of the results. This figure shows that the effects of distinctiveness (distinctive > typical) tend to occur in overlapping regions for both stimulus types (note that, with an uncorrected threshold, the clusters related to chair distinctiveness overlap with those related to face distinctiveness). Conversely, typicality effects seem to be differently distributed and more prominent for chairs. (TIF) [file pone.0293781.s005.tif]
