# Supplementary material for: Investigating the neural effects of typicality and predictability for face and object stimuli
Source: PLoS One. 2024 May 22;19(5):e0293781. doi: 10.1371/journal.pone.0293781 (PMC11111078; doi:10.1371/journal.pone.0293781)
Supplement: S1 File — Labels assigned to the significant activation clusters by means of the Anatomy Toolbox (https://www.fz-juelich.de/en/inm/inm-7/resources/jubrain-anatomy-toolbox). Whole-brain maps resulting from our contrasts of interest were entered in the toolbox. The contrast as well as the coordinates of maximal activation, the size of the activation cluster and the brain regions most likely included in the cluster are reported in the file. This file is provided separately. (PDF) [file pone.0293781.s006.pdf]

Raw output of the Anatomy Toolbox

https://www.fz-juelich.de/en/inm/inm-7/resources/jubrain-anatomy-toolbox

t-contrast Chairs > Faces (p_{FWE}= .05)

Image: MAIN_t_Chairs-Faces_FWE05 [u=0.0, k=0]

Cluster 1 2763 vox +26 -52 -10

Assignment based on Maximum Probability Map Percent of Cluster volume in Area Percent of Area activated by Cluster

Area hOc4la	9.9	16.0
Area hOc4lp	9.6	18.4
Area hOc4v [V4(v)]	6.8	13.7
Area hIP7 (IPS)	5.6	31.7
Area hIP4 (IPS)	4.4	29.7
Area FG3	3.5	11.4
Area FG1	2.6	12.2
Area hOc3v [V3v]	2.4	4.0
Area hIP8 (IPS)	2.3	10.5
Area hOc4d [V3A]	1.4	3.9
Area hOc3d [V3d]	0.8	1.7
Area hPO1 (IPS)	0.7	2.9
CA1 (Hippocampus)	0.7	1.9
Area PGp (IPL)	0.5	0.8
Area hIP5 (IPS)	0.2	1.0
Area hOc1 [V1]	0.2	0.2
Area hOc5 [V5/MT]	0.1	1.4
Area 7P (SPL)	0.0	0.1
Subiculum	0.0	0.1

DG (Hippocampus)	0.0	0.0

Probability exceedance by Area	Mean probability at Cluster location (%)	Mean probability across PMap (%)	Ratio	95% CI	95% CI
Area hOc4la	37.5	30.4	1.23	1.20	1.26
Area hOc4lp	28.1	25.6	1.10	1.07	1.12
Area hOc4v [V4(v)]	29.7	25.3	1.17	1.14	1.19
Area hIP7 (IPS)	27.0	17.4	1.55	1.50	1.60
Area FG3	24.2	23.6	1.03	0.99	1.06
Area hIP4 (IPS)	18.1	17.0	1.07	1.02	1.10
Area hOc3v [V3v]	21.8	25.2	0.87	0.84	0.89
Area FG1	17.7	18.9	0.94	0.90	0.97
Area hIP8 (IPS)	23.6	18.9	1.25	1.20	1.29
Area hOc4d [V3A]	14.0	20.1	0.69	0.67	0.72
Area hPO1 (IPS)	9.1	19.2	0.48	0.45	0.50
Area hOc3d [V3d]	10.1	21.3	0.48	0.46	0.50
Area PGp (IPL)	11.4	25.1	0.45	0.43	0.48
CA1 (Hippocampus)	22.1	23.5	0.94	0.88	1.00
Area hIP5 (IPS)	12.0	20.9	0.57	0.53	0.61
Area hOc1 [V1]	10.0	34.8	0.29	0.26	0.31
Area hOc5 [V5/MT]	7.3	15.0	0.49	0.45	0.53
Area hOc2 [V2]	2.6	20.6	0.13	0.12	0.14
Area 7P (SPL)	9.2	22.4	0.41	0.38	0.45
DG (Hippocampus)	8.2	16.2	0.51	0.44	0.59
Subiculum	8.2	21.4	0.38	0.32	0.44
Area FG2	2.7	20.1	0.14	0.12	0.15
Area hIP6 (IPS)	6.7	19.1	0.35	0.28	0.42
Area FG4	2.0	24.1	0.08	0.07	0.09
Area 7A (SPL)	2.7	24.2	0.11	0.10	0.12
Area hOc6 [V6]	1.7	20.1	0.08	0.07	0.10

Top probabilites at peak voxels	Union across peaks
Area hOc4la	0.6
Area hOc4lp	0.6
Area hOc4v [V4(v)]	0.5
Area hOc3v [V3v]	0.5
Area hIP8 (IPS)	0.5
Area hPO1 (IPS)	0.3
Area hIP4 (IPS)	0.3
Area 7P (SPL)	0.1
Area FG3	0.1
Area hOc2 [V2]	0.1
Area hIP7 (IPS)	0.0
Area hIP5 (IPS)	0.0
Area 7A (SPL)	0.0
Area FG2	0.0
Area hOc5 [V5/MT]	0.0

Probabilities at local maxima +26.0 / -52.0 / -10.0 (Height: 14) +42.0 / -80.0 / +4.0 (Height: 14) +32.0 / -84.0 / +14.0 (Height: 13) +22.0 / -76.0 / -10.0 (Height: 8.9) +18.0 / -78.0 / +44.0 (Height: 7.2) +20.0 / -76.0 / +42.0 (Height: 7.2) +48.0 / -58.0 / -6.0 (Height: 6.4)

Area hOc4la	0.0	62.7	2.0	0.0	0.0	0.0	0.0
Area hOc4lp	0.0	37.2	32.1	0.0	0.0	0.0	0.0
Area hOc4v [V4(v)]	0.0	0.0	0.0	47.6	0.0	0.0	0.0
Area hOc3v [V3v]	0.0	0.0	0.0	47.3	0.0	0.0	0.0
Area hIP8 (IPS)	0.0	0.0	0.0	0.0	19.5	32.1	0.0
Area hPO1 (IPS)	0.0	0.0	0.0	0.0	6.0	24.3	0.0
Area hIP4 (IPS)	0.0	0.0	28.3	0.0	0.0	0.0	0.0
Area 7P (SPL)	0.0	0.0	0.0	0.0	11.5	3.3	0.0
Area FG3	11.4	0.0	0.0	0.0	0.0	0.0	0.0
Area hOc2 [V2]	0.0	0.0	0.0	5.1	0.0	0.0	0.0

Area hIP7 (IPS)	0.0	0.0	4.5	0.0	0.0	0.0	0.0
Area hIP5 (IPS)	0.0	0.0	0.0	0.0	0.0	3.2	0.0
Area 7A (SPL)	0.0	0.0	0.0	0.0	0.3	0.0	0.0
Area FG2	0.0	0.0	0.0	0.0	0.0	0.0	0.2
Area hOc5 [V5/MT]	0.0	0.1	0.0	0.0	0.0	0.0	0.0

Reference List

Area hOc6 [V6]

M. Richter, K. Amunts, H. Mohlberg, S. Bludau, S.B. Eickhoff, K. Zilles, S. Caspers. Cytoarchitectonic segregation of human posterior intraparietal and adjacent parieto-occipital sulcus and ist relation to visuomotor and cognitive functions. Cereb Cortex, in press, 2018

CA1 (Hippocampus)

K. Amunts, O. Kedo., M. Kindler., P. Pieperhoff., F. Schneider, H. Mohlberg, U. Habel, J.N. Shah, K. Zilles. Cytoarchitectonic mapping of the human amygdala, hippocampal region and entorhinal cortex. Anatomy and Embryology 210 (5-6): 343-352, 2005

Area hOc4v [V4(v)]

C. Rottschy, S.B. Eickhoff, A. Schleicher, H. Mohlberg, K. Zilles, K. Amunts. The ventral visual cortex in humans: Cytoarchitectonic mapping of two extrastriate areas. Human Brain Mapping 28 (1): 1-8, 2007

Area FG2

J. Caspers, K. Zilles, S.B. Eickhoff, A. Schleicher, H. Mohlberg, K. Amunts. Cytoarchitectonical analysis and probabilistic mapping of two extrastriate areas of the human posterior fusiform gyrus. Brain Structure and Function 218: 511-526, 2013.

Area hIP5 (IPS)

M. Richter, K. Amunts, H. Mohlberg, S. Bludau, S.B. Eickhoff, K. Zilles, S. Caspers. Cytoarchitectonic segregation of human posterior intraparietal and adjacent parieto-occipital sulcus and ist relation to visuomotor and cognitive functions. Cereb Cortex, in press, 2018

Area hOc4d [V3A]

M. Kujovic, K. Zilles, A. Malikovic, A. Schleicher, H. Mohlberg, C. Rottschy, S.B. Eickhoff, K. Amunts. Cytoarchitectonic mapping of the human dorsal extrastriate cortex. Brain Struct. Funct. 218 (1): 157-172, 2013.

Area hOc2 [V2]

K. Amunts, A. Malikovic, H.Mohlberg, T.Schormann, K. Zilles. Brodmann's areas 17 and 18 brought into stereotaxic space - where and how variable? NeuroImage 11: 66-84, 2000

Area 7P (SPL)

F. Scheperjans, K. Hermann, S. B. Eickhoff, K. Amunts, A. Schleicher, K. Zilles. Observer-independent cytoarchitectonic mapping of the human superior parietal cortex. Cereb.Cortex 18 (4): 846-867, 2008.

F. Scheperjans, S. B. Eickhoff, L. Hömke, H. Mohlberg, K. Hermann, K. Amunts, K. Zilles. Probabilistic maps, morphometry, and variability of cytoarchitectonic areas in the human superior parietal cortex. Cereb.Cortex 18 (9): 2141-2157, 2008.

Area hOc4la

A. Malikovic, K. Amunts, A. Schleicher, H. Mohlberg, M. Kujovic, S.E. Eickhoff, K. Zilles. Cytoarchitecture of the human lateral occipital cortex: Mapping of two extrastriate areas hOc4la and hOc4lp. Brain Structure and Function, 221(4):1877-97, 2016.

Area hOc5 [V5/MT]

A. Malikovic, K. Amunts, A. Schleicher, H. Mohlberg, S.B. Eickhoff, M.Wilms, N. Palomero-Gallagher, E. Armstrong, K. Zilles. Cytoarchitectonic analysis of the human extrastriate cortex in the region of V5/MT+: A probabilistic, stereotaxic map of area hOc5, Cereb Cortex 17 (3): 562-574, 2007

Area hOc3v [V3v]

C. Rottschy, S.B. Eickhoff, A. Schleicher, H. Mohlberg, K. Zilles, K. Amunts. The ventral visual cortex in humans: Cytoarchitectonic mapping of two extrastriate areas. Human Brain Mapping 28 (1): 1-8, 2007

Area 7A (SPL)

F. Scheperjans, K. Hermann, S. B. Eickhoff, K. Amunts, A. Schleicher, K. Zilles. Observer-independent cytoarchitectonic mapping of the human superior parietal cortex. Cereb.Cortex 18 (4): 846-867, 2008.

F. Scheperjans, S. B. Eickhoff, L. Hömke, H. Mohlberg, K. Hermann, K. Amunts, K. Zilles. Probabilistic maps, morphometry, and variability of cytoarchitectonic areas in the human superior parietal cortex. Cereb.Cortex 18 (9): 2141-2157, 2008.

Area hIP7 (IPS)

M. Richter, K. Amunts, H. Mohlberg, S. Bludau, S.B. Eickhoff, K. Zilles, S. Caspers. Cytoarchitectonic segregation of human posterior intraparietal and adjacent parieto-occipital sulcus and ist relation to visuomotor and cognitive functions. Cereb Cortex, in press, 2018

Area hOc1 [V1]

K. Amunts, A. Malikovic, H.Mohlberg, T.Schormann, K. Zilles. Brodmann's areas 17 and 18 brought into stereotaxic space - where and how variable? NeuroImage 11: 66-84, 2000

Area hOc3d [V3d]

M. Kujovic, K. Zilles, A. Malikovic, A. Schleicher, H. Mohlberg, C. Rottschy, S.B. Eickhoff, K. Amunts. Cytoarchitectonic mapping of the human dorsal extrastriate cortex. Brain Struct. Funct. 218 (1): 157-172, 2013.

Area hIP8 (IPS)

M. Richter, K. Amunts, H. Mohlberg, S. Bludau, S.B. Eickhoff, K. Zilles, S. Caspers. Cytoarchitectonic segregation of human posterior intraparietal and adjacent parieto-occipital sulcus and ist relation to visuomotor and cognitive functions. Cereb Cortex, in press, 2018

Area FG4

S. Lorenz, K.S. Weiner, J. Caspers, H. Mohlberg, A. Schleicher, S. Bludau, S.B. Eickhoff, K. Grill-Spector, K. Zilles, K. Amunts. Two new cytoarchitectonic areas on the human mid-fusiform gyrus. Cerebral Cortex 27 (1): 373-385, 2017.

Area FG3

S. Lorenz, K.S. Weiner, J. Caspers, H. Mohlberg, A. Schleicher, S. Bludau, S.B. Eickhoff, K. Grill-Spector, K. Zilles, K. Amunts. Two new cytoarchitectonic areas on the human mid-fusiform gyrus. Cerebral Cortex 27 (1): 373-385, 2017.

Subiculum

K. Amunts, O. Kedo., M. Kindler., P. Pieperhoff., F. Schneider, H. Mohlberg, U. Habel, J.N. Shah, K. Zilles. Cytoarchitectonic mapping of the human amygdala, hippocampal region and entorhinal cortex. Anatomy and Embryology 210 (5-6): 343-352, 2005

DG (Hippocampus)

K. Amunts, O. Kedo., M. Kindler., P. Pieperhoff., F. Schneider, H. Mohlberg, U. Habel, J.N. Shah, K. Zilles. Cytoarchitectonic mapping of the human amygdala, hippocampal region and entorhinal cortex. Anatomy and Embryology 210 (5-6): 343-352, 2005

Area hOc4lp

A. Malikovic, K. Amunts, A. Schleicher, H. Mohlberg, M. Kujovic, S.E. Eickhoff, K. Zilles. Cytoarchitecture of the human lateral occipital cortex: Mapping of two extrastriate areas hOc4la and hOc4lp. Brain Structure and Function, 221(4):1877-97, 2016.

Area hIP4 (IPS)

M. Richter, K. Amunts, H. Mohlberg, S. Bludau, S.B. Eickhoff, K. Zilles, S. Caspers. Cytoarchitectonic segregation of human posterior intraparietal and adjacent parieto-occipital sulcus and ist relation to visuomotor and cognitive functions. Cereb Cortex, in press, 2018

Area FG1

J. Caspers, K. Zilles, S.B. Eickhoff, A. Schleicher, H. Mohlberg, K. Amunts. Cytoarchitectonical analysis and probabilistic mapping of two extrastriate areas of the human posterior fusiform gyrus. Brain Structure and Function 218: 511-526, 2013.

Area PGp (IPL)

S. Caspers, S. Geyer, A. Schleicher, H. Mohlberg, K. Amunts, K. Zilles. The human inferior parietal cortex: Cytoarchitectonic parcellation and interindividual variability. Neuroimage 33 (2): 430-448, 2006.

S. Caspers, S. B. Eickhoff, S. Geyer, F. Scheperjans, H. Mohlberg, K. Zilles, K. Amunts. The human inferior parietal lobule in stereotaxic space. Brain Structure and Function 212 (6): 481-495, 2008.

Area hIP6 (IPS)

M. Richter, K. Amunts, H. Mohlberg, S. Bludau, S.B. Eickhoff, K. Zilles, S. Caspers. Cytoarchitectonic segregation of human posterior intraparietal and adjacent parieto-occipital sulcus and ist relation to visuomotor and cognitive functions. Cereb Cortex, in press, 2018

Area hPO1 (IPS)

M. Richter, K. Amunts, H. Mohlberg, S. Bludau, S.B. Eickhoff, K. Zilles, S. Caspers. Cytoarchitectonic segregation of human posterior intraparietal and adjacent parieto-occipital sulcus and ist relation to visuomotor and cognitive functions. Cereb Cortex, in press, 2018

Cluster 2 2542 vox -36 -88 +8

Assignment based on Maximum Probability Map	Percent of Cluster volume in Area	Percent of Area activated by Cluster
Area hOc4lp	10.3	18.2
Area hOc4la	7.4	10.9
Area hOc4v [V4(v)]	7.2	13.5
Area FG3	6.1	18.1
Area hOc4d [V3A]	3.2	8.5
Area hIP7 (IPS)	3.1	16.1
Area FG1	2.9	12.4
Area hOc5 [V5/MT]	2.5	36.9
Area hIP4 (IPS)	1.9	12.0
Area hOc3v [V3v]	1.8	2.8
Area hOc1 [V1]	1.1	0.9
Area hOc3d [V3d]	1.0	2.0
CA1 (Hippocampus)	0.9	2.3
Area FG2	0.2	0.6
Subiculum	0.2	0.6
Area hIP5 (IPS)	0.1	0.3
Area hOc2 [V2]	0.1	0.1
Area hIP8 (IPS)	0.1	0.3
Area FG4	0.0	0.1

Area hPO1 (IPS)	0.0	0.0

Probability exceedance by Area	Mean probability at Cluster location (%)	Mean probability across PMap (%)	Ratio	95% CI	95% CI

Area hOc4lp	30.1	25.6	1.18	1.15	1.20
Area hOc4la	28.6	30.4	0.94	0.92	0.96
Area FG3	29.3	23.6	1.24	1.21	1.28
Area hOc4v [V4(v)]	31.1	25.3	1.23	1.20	1.26
Area hOc3v [V3v]	18.5	25.2	0.73	0.71	0.75
Area FG1	18.6	18.9	0.98	0.95	1.03
Area hOc4d [V3A]	18.4	20.1	0.92	0.88	0.95
Area hIP4 (IPS)	22.3	17.0	1.32	1.27	1.36
Area hOc5 [V5/MT]	24.3	15.0	1.62	1.55	1.70
Area hIP7 (IPS)	21.3	17.4	1.23	1.17	1.29
Area hOc3d [V3d]	14.9	21.3	0.70	0.67	0.73
Area hOc1 [V1]	14.7	34.8	0.42	0.40	0.45
CA1 (Hippocampus)	21.7	23.5	0.92	0.87	0.98
Area FG2	8.0	20.1	0.40	0.38	0.42
Area hOc2 [V2]	4.7	20.6	0.23	0.21	0.24
Area FG4	6.0	24.1	0.25	0.23	0.27
Subiculum	14.1	21.4	0.66	0.59	0.74
Area hIP8 (IPS)	9.0	18.9	0.48	0.42	0.53
Area hPO1 (IPS)	5.8	19.2	0.30	0.28	0.33
DG (Hippocampus)	8.6	16.2	0.53	0.46	0.60
Area hIP5 (IPS)	9.8	20.9	0.47	0.38	0.57
Area PGp (IPL)	6.2	25.1	0.25	0.19	0.30
Area hOc6 [V6]	1.6	20.1	0.08	0.05	0.11

Top probabilites at peak voxels	Union across peaks
Area hOc4v [V4(v)]	0.9
Area FG3	0.9

Area hOc4lp	0.8
Area hOc3v [V3v]	0.6
Area hOc4la	0.5
Area hOc5 [V5/MT]	0.5
Area FG2	0.1
Area FG4	0.1
Area FG1	0.1
CA1 (Hippocampus)	0.0
Area hOc1 [V1]	0.0

Probabilities at local maxima -36.0 / -88.0 / +8.0 (Height: 14) -30.0 / -44.0 / -12.0 (Height: 12) -32.0 / -46.0 / -10.0 (Height: 12) -26.0 / -58.0 / -8.0 (Height: 11) -42.0 / -78.0 / -2.0 (Height: 8.8) -30.0 / -74.0 / +0.0 (Height: 7.9) -44.0 / -62.0 / -4.0 (Height: 7.5) -20.0 / -76.0 / -10.0 (Height: 7.4) -22.0 / -78.0 / -8.0 (Height: 7.4)

Area hOc4v [V4(v)]	0.0	0.0	0.0	0.0	0.0	0.1	0.0	66.5	65.3
Area FG3	0.0	38.1	71.3	32.5	0.0	0.0	0.0	0.0	0.0
Area hOc4lp	76.4	0.0	0.0	0.0	0.0	0.0	0.0	0.0	0.0
Area hOc3v [V3v]	0.0	0.0	0.0	0.0	0.0	0.0	0.0	33.1	34.2
Area hOc4la	23.6	0.0	0.0	0.0	35.8	0.0	0.0	0.0	0.0
Area hOc5 [V5/MT]	0.0	0.0	0.0	0.0	50.3	0.0	0.0	0.0	0.0
Area FG2	0.0	0.0	0.0	0.0	13.9	0.0	0.0	0.0	0.0
Area FG4	0.0	0.0	0.0	0.0	0.0	0.0	8.2	0.0	0.0
Area FG1	0.0	0.0	0.0	0.0	0.0	5.8	0.0	0.5	0.5
CA1 (Hippocampus)	0.0	3.1	0.0	0.0	0.0	0.0	0.0	0.0	0.0
Area hOc1 [V1]	0.0	0.0	0.0	0.8	0.0	0.0	0.0	0.0	0.0

Reference List

Area hOc6 [V6]

M. Richter, K. Amunts, H. Mohlberg, S. Bludau, S.B. Eickhoff, K. Zilles, S. Caspers. Cytoarchitectonic segregation of human posterior intraparietal and adjacent parieto-occipital sulcus and ist relation to visuomotor and cognitive functions. Cereb Cortex, in press, 2018

CA1 (Hippocampus)

K. Amunts, O. Kedo., M. Kindler., P. Pieperhoff., F. Schneider, H. Mohlberg, U. Habel, J.N. Shah, K. Zilles. Cytoarchitectonic mapping of the human amygdala, hippocampal region and entorhinal cortex. Anatomy and Embryology 210 (5-6): 343-352, 2005

Area hOc4v [V4(v)]

C. Rottschy, S.B. Eickhoff, A. Schleicher, H. Mohlberg, K. Zilles, K. Amunts. The ventral visual cortex in humans: Cytoarchitectonic mapping of two extrastriate areas. Human Brain Mapping 28 (1): 1-8, 2007

Area FG2

J. Caspers, K. Zilles, S.B. Eickhoff, A. Schleicher, H. Mohlberg, K. Amunts. Cytoarchitectonical analysis and probabilistic mapping of two extrastriate areas of the human posterior fusiform gyrus. Brain Structure and Function 218: 511-526, 2013.

Area hIP5 (IPS)

M. Richter, K. Amunts, H. Mohlberg, S. Bludau, S.B. Eickhoff, K. Zilles, S. Caspers. Cytoarchitectonic segregation of human posterior intraparietal and adjacent parieto-occipital sulcus and ist relation to visuomotor and cognitive functions. Cereb Cortex, in press, 2018

Area hOc4d [V3A]

M. Kujovic, K. Zilles, A. Malikovic, A. Schleicher, H. Mohlberg, C. Rottschy, S.B. Eickhoff, K. Amunts. Cytoarchitectonic mapping of the human dorsal extrastriate cortex. Brain Struct. Funct. 218 (1): 157-172, 2013.

Area hOc2 [V2]

K. Amunts, A. Malikovic, H.Mohlberg, T.Schormann, K. Zilles. Brodmann's areas 17 and 18 brought into stereotaxic space - where and how variable? NeuroImage 11: 66-84, 2000

Area hOc4la

A. Malikovic, K. Amunts, A. Schleicher, H. Mohlberg, M. Kujovic, S.E. Eickhoff, K. Zilles. Cytoarchitecture of the human lateral occipital cortex: Mapping of two extrastriate areas hOc4la and hOc4lp. Brain Structure and Function, 221(4):1877-97, 2016.

Area hOc5 [V5/MT]

A. Malikovic, K. Amunts, A. Schleicher, H. Mohlberg, S.B. Eickhoff, M.Wilms, N. Palomero-Gallagher, E. Armstrong, K. Zilles. Cytoarchitectonic analysis of the human extrastriate cortex in the region of V5/MT+: A probabilistic, stereotaxic map of area hOc5, Cereb Cortex 17 (3): 562-574, 2007

Area hOc3v [V3v]

C. Rottschy, S.B. Eickhoff, A. Schleicher, H. Mohlberg, K. Zilles, K. Amunts. The ventral visual cortex in humans: Cytoarchitectonic mapping of two extrastriate areas. Human Brain Mapping 28 (1): 1-8, 2007

Area hIP7 (IPS)

M. Richter, K. Amunts, H. Mohlberg, S. Bludau, S.B. Eickhoff, K. Zilles, S. Caspers. Cytoarchitectonic segregation of human posterior intraparietal and adjacent parieto-occipital sulcus and ist relation to visuomotor and cognitive functions. Cereb Cortex, in press, 2018

Area hOc1 [V1]

K. Amunts, A. Malikovic, H.Mohlberg, T.Schormann, K. Zilles. Brodmann's areas 17 and 18 brought into stereotaxic space - where and how variable? NeuroImage 11: 66-84, 2000

Area hOc3d [V3d]

M. Kujovic, K. Zilles, A. Malikovic, A. Schleicher, H. Mohlberg, C. Rottschy, S.B. Eickhoff, K. Amunts. Cytoarchitectonic mapping of the human dorsal extrastriate cortex. Brain Struct. Funct. 218 (1): 157-172, 2013.

Area hIP8 (IPS)

M. Richter, K. Amunts, H. Mohlberg, S. Bludau, S.B. Eickhoff, K. Zilles, S. Caspers. Cytoarchitectonic segregation of human posterior intraparietal and adjacent parieto-occipital sulcus and ist relation to visuomotor and cognitive functions. Cereb Cortex, in press, 2018

Area FG4

S. Lorenz, K.S. Weiner, J. Caspers, H. Mohlberg, A. Schleicher, S. Bludau, S.B. Eickhoff, K. Grill-Spector, K. Zilles, K. Amunts. Two new cytoarchitectonic areas on the human mid-fusiform gyrus. Cerebral Cortex 27 (1): 373-385, 2017.

Area FG3

S. Lorenz, K.S. Weiner, J. Caspers, H. Mohlberg, A. Schleicher, S. Bludau, S.B. Eickhoff, K. Grill-Spector, K. Zilles, K. Amunts. Two new cytoarchitectonic areas on the human mid-fusiform gyrus. Cerebral Cortex 27 (1): 373-385, 2017.

Subiculum

K. Amunts, O. Kedo., M. Kindler., P. Pieperhoff., F. Schneider, H. Mohlberg, U. Habel, J.N. Shah, K. Zilles. Cytoarchitectonic mapping of the human amygdala, hippocampal region and entorhinal cortex. Anatomy and Embryology 210 (5-6): 343-352, 2005

DG (Hippocampus)

K. Amunts, O. Kedo., M. Kindler., P. Pieperhoff., F. Schneider, H. Mohlberg, U. Habel, J.N. Shah, K. Zilles. Cytoarchitectonic mapping of the human amygdala, hippocampal region and entorhinal cortex. Anatomy and Embryology 210 (5-6): 343-352, 2005

Area hOc4lp

A. Malikovic, K. Amunts, A. Schleicher, H. Mohlberg, M. Kujovic, S.E. Eickhoff, K. Zilles. Cytoarchitecture of the human lateral occipital cortex: Mapping of two extrastriate areas hOc4la and hOc4lp. Brain Structure and Function, 221(4):1877-97, 2016.

Area hIP4 (IPS)

M. Richter, K. Amunts, H. Mohlberg, S. Bludau, S.B. Eickhoff, K. Zilles, S. Caspers. Cytoarchitectonic segregation of human posterior intraparietal and adjacent parieto-occipital sulcus and ist relation to visuomotor and cognitive functions. Cereb Cortex, in press, 2018

Area FG1

J. Caspers, K. Zilles, S.B. Eickhoff, A. Schleicher, H. Mohlberg, K. Amunts. Cytoarchitectonical analysis and probabilistic mapping of two extrastriate areas of the human posterior fusiform gyrus. Brain Structure and Function 218: 511-526, 2013.

Area PGp (IPL)

S. Caspers, S. Geyer, A. Schleicher, H. Mohlberg, K. Amunts, K. Zilles. The human inferior parietal cortex: Cytoarchitectonic parcellation and interindividual variability. Neuroimage 33 (2): 430-448, 2006.

S. Caspers, S. B. Eickhoff, S. Geyer, F. Scheperjans, H. Mohlberg, K. Zilles, K. Amunts. The human inferior parietal lobule in stereotaxic space. Brain Structure and Function 212 (6): 481-495, 2008.

Area hPO1 (IPS)

M. Richter, K. Amunts, H. Mohlberg, S. Bludau, S.B. Eickhoff, K. Zilles, S. Caspers. Cytoarchitectonic segregation of human posterior intraparietal and adjacent parieto-occipital sulcus and ist relation to visuomotor and cognitive functions. Cereb Cortex, in press, 2018

Cluster 3 32 vox +18 -62 +52

Assignment based on Maximum Probability Map	Percent of Cluster volume in Area	Percent of Area activated by Cluster
Area 7A (SPL)	33.2	0.6
Area hIP3 (IPS)	10.2	0.3
Area 7P (SPL)	3.9	0.2

Probability exceedance by Area	Mean probability at Cluster location (%)	Mean probability across PMap (%)	Ratio	95% CI	95% CI
Area 7A (SPL)	28.0	24.2	1.15	1.07	1.23
Area hIP3 (IPS)	16.5	18.2	0.90	0.79	1.01
Area 7P (SPL)	14.2	22.4	0.63	0.58	0.70
Area 5L (SPL)	5.1	20.1	0.25	0.14	0.38
Area 7PC (SPL)	3.7	17.1	0.22	0.10	0.34

Top probabilites at peak voxels	Union across peaks
Area 7A (SPL)	0.5
Area hIP3 (IPS)	0.2
Area 7P (SPL)	0.0
Area 5L (SPL)	0.0

Area 7PC (SPL)	0.0

Probabilities at local maxima +18.0 / -62.0 / +52.0 (Height: 6.3) +22.0 / -56.0 / +56.0 (Height: 5.6)

Area 7A (SPL)	28.6	36.2
Area hIP3 (IPS)	15.1	3.1
Area 7P (SPL)	4.6	0.0
Area 5L (SPL)	0.0	2.5
Area 7PC (SPL)	0.0	0.6

Reference List

Area 5L (SPL)

F. Scheperjans, K. Hermann, S. B. Eickhoff, K. Amunts, A. Schleicher, K. Zilles. Observer-independent cytoarchitectonic mapping of the human superior parietal cortex. Cereb.Cortex 18 (4): 846-867, 2008.

F. Scheperjans, S. B. Eickhoff, L. Hömke, H. Mohlberg, K. Hermann, K. Amunts, K. Zilles. Probabilistic maps, morphometry, and variability of cytoarchitectonic areas in the human superior parietal cortex. Cereb.Cortex 18 (9): 2141-2157, 2008.

Area 7P (SPL)

F. Scheperjans, K. Hermann, S. B. Eickhoff, K. Amunts, A. Schleicher, K. Zilles. Observer-independent cytoarchitectonic mapping of the human superior parietal cortex. Cereb.Cortex 18 (4): 846-867, 2008.

F. Scheperjans, S. B. Eickhoff, L. Hömke, H. Mohlberg, K. Hermann, K. Amunts, K. Zilles. Probabilistic maps, morphometry, and variability of cytoarchitectonic areas in the human superior parietal cortex. Cereb.Cortex 18 (9): 2141-2157, 2008.

Area 7A (SPL)

F. Scheperjans, K. Hermann, S. B. Eickhoff, K. Amunts, A. Schleicher, K. Zilles. Observer-independent cytoarchitectonic mapping of the human superior parietal cortex. Cereb.Cortex 18 (4): 846-867, 2008.

F. Scheperjans, S. B. Eickhoff, L. Hömke, H. Mohlberg, K. Hermann, K. Amunts, K. Zilles. Probabilistic maps, morphometry, and variability of cytoarchitectonic areas in the human superior parietal cortex. Cereb.Cortex 18 (9): 2141-2157, 2008.

Area hIP3 (IPS)

F. Scheperjans, K. Hermann, S. B. Eickhoff, K. Amunts, A. Schleicher, K. Zilles. Observer-independent cytoarchitectonic mapping of the human superior parietal cortex. Cereb.Cortex 18 (4): 846-867, 2008.

F. Scheperjans, S. B. Eickhoff, L. Hömke, H. Mohlberg, K. Hermann, K. Amunts, K. Zilles. Probabilistic maps, morphometry, and variability of cytoarchitectonic areas in the human superior parietal cortex. Cereb.Cortex 18 (9): 2141-2157, 2008.

Area 7PC (SPL)

F. Scheperjans, K. Hermann, S. B. Eickhoff, K. Amunts, A. Schleicher, K. Zilles. Observer-independent cytoarchitectonic mapping of the human superior parietal cortex. Cereb.Cortex 18 (4): 846-867, 2008.

F. Scheperjans, S. B. Eickhoff, L. Hömke, H. Mohlberg, K. Hermann, K. Amunts, K. Zilles. Probabilistic maps, morphometry, and variability of cytoarchitectonic areas in the human superior parietal cortex. Cereb.Cortex 18 (9): 2141-2157, 2008.

t-contrast Faces > Chairs (p_{FWE}= .05)

Image: MAIN_t_Faces-Chairs_FWE05 [u=0.0, k=0]

Cluster 1 64 vox +54 -50 +8

Assignment based on Maximum Probability Map Percent of Cluster volume in Area Percent of Area activated by Cluster

Probability exceedance by Area Mean probability at Cluster location (%) Mean probability across PMap (%) Ratio 95% CI 95% CI

Area PGa (IPL)	4.6	20.5	0.23	0.19	0.25
Area PFm (IPL)	0.6	21.7	0.03	0.00	0.06

Area PGp (IPL)	1.1	25.1	0.04	0.01	0.09

Top probabilites at peak voxels Union across peaks

Area PGa (IPL) 0.0

Probabilities at local maxima +54.0 / -50.0 / +8.0 (Height: 6.5)

Area PGa (IPL) 3.1

Reference List

Area PGa (IPL)

S. Caspers, S. Geyer, A. Schleicher, H. Mohlberg, K. Amunts, K. Zilles. The human inferior parietal cortex: Cytoarchitectonic parcellation and interindividual variability. Neuroimage 33 (2): 430-448, 2006.

S. Caspers, S. B. Eickhoff, S. Geyer, F. Scheperjans, H. Mohlberg, K. Zilles, K. Amunts. The human inferior parietal lobule in stereotaxic space. Brain Structure and Function 212 (6): 481-495, 2008.

Area PFm (IPL)

S. Caspers, S. Geyer, A. Schleicher, H. Mohlberg, K. Amunts, K. Zilles. The human inferior parietal cortex: Cytoarchitectonic parcellation and interindividual variability. Neuroimage 33 (2): 430-448, 2006.

S. Caspers, S. B. Eickhoff, S. Geyer, F. Scheperjans, H. Mohlberg, K. Zilles, K. Amunts. The human inferior parietal lobule in stereotaxic space. Brain Structure and Function 212 (6): 481-495, 2008.

Area PGp (IPL)

S. Caspers, S. Geyer, A. Schleicher, H. Mohlberg, K. Amunts, K. Zilles. The human inferior parietal cortex: Cytoarchitectonic parcellation and interindividual variability. Neuroimage 33 (2): 430-448, 2006.

S. Caspers, S. B. Eickhoff, S. Geyer, F. Scheperjans, H. Mohlberg, K. Zilles, K. Amunts. The human inferior parietal lobule in stereotaxic space. Brain Structure and Function 212 (6): 481-495, 2008.

t-contrast Distinctive > Typical (p_{FWE}= .05)

Image: MAIN_t_Dist-Typ_FWE05 [u=0.0, k=0]

Cluster 1 293 vox +34 -80 -10

Assignment based on Maximum Probability Map Percent of Cluster volume in Area Percent of Area activated by Cluster

Area hOc4la 49.2 8.4

Area hOc4v [V4(v)] 29.6 6.4

Area hOc4lp 10.2 2.1

Area hOc3v [V3v] 5.6 1.0

Area FG2 1.9 0.7

Area FG1 1.2 0.6

Probability exceedance by Area Mean probability at Cluster location (%) Mean probability across PMap (%) Ratio 95% CI 95% CI

Area hOc4la 47.4 30.4 1.56 1.52 1.61

Area hOc4v [V4(v)] 29.1 25.3 1.15 1.11 1.19

Area hOc4lp 18.5 25.6 0.72 0.69 0.75

Area FG1 15.6 18.9 0.83 0.79 0.86

Area hOc3v [V3v] 23.5 25.2 0.93 0.85 1.00

Area FG2 11.7 20.1 0.58 0.55 0.62

Area hOc2 [V2] 3.0 20.6 0.15 0.10 0.19

Area hOc5 [V5/MT] 1.1 15.0 0.07 0.05 0.10

Top probabilites at peak voxels Union across peaks

Area hOc4la 1.0

Area hOc4v [V4(v)] 0.9

Area FG1 0.5

Area hOc4lp	0.3
Area FG2	0.1
Area hOc3v [V3v]	0.0
Area hOc5 [V5/MT]	0.0

Probabilities at local maxima +34.0 / -80.0 / -10.0 (Height: 7.8) +44.0 / -80.0 / +0.0 (Height: 7.6) +36.0 / -78.0 / -12.0 (Height: 7.6) +42.0 / -78.0 / -2.0 (Height: 7.6)

Area hOc4la	0.0	82.2	0.0	74.6
Area hOc4v [V4(v)]	65.1	0.0	61.8	0.0
Area FG1	22.7	0.0	31.5	0.0
Area hOc4lp	3.3	17.7	0.7	10.7
Area FG2	0.0	0.0	6.0	0.0
Area hOc3v [V3v]	4.6	0.0	0.0	0.0
Area hOc5 [V5/MT]	0.0	0.0	0.0	0.0

Reference List

Area hOc4v [V4(v)]

C. Rottschy, S.B. Eickhoff, A. Schleicher, H. Mohlberg, K. Zilles, K. Amunts. The ventral visual cortex in humans: Cytoarchitectonic mapping of two extrastriate areas. Human Brain Mapping 28 (1): 1-8, 2007

Area FG2

J. Caspers, K. Zilles, S.B. Eickhoff, A. Schleicher, H. Mohlberg, K. Amunts. Cytoarchitectonical analysis and probabilistic mapping of two extrastriate areas of the human posterior fusiform gyrus. Brain Structure and Function 218: 511-526, 2013.

Area hOc2 [V2]

K. Amunts, A. Malikovic, H.Mohlberg, T.Schormann, K. Zilles. Brodmann's areas 17 and 18 brought into stereotaxic space - where and how variable? NeuroImage 11: 66-84, 2000

Area hOc4la

A. Malikovic, K. Amunts, A. Schleicher, H. Mohlberg, M. Kujovic, S.E. Eickhoff, K. Zilles. Cytoarchitecture of the human lateral occipital cortex: Mapping of two extrastriate areas hOc4la and hOc4lp. Brain Structure and Function, 221(4):1877-97, 2016.

Area hOc5 [V5/MT]

A. Malikovic, K. Amunts, A. Schleicher, H. Mohlberg, S.B. Eickhoff, M.Wilms, N. Palomero-Gallagher, E. Armstrong, K. Zilles. Cytoarchitectonic analysis of the human extrastriate cortex in the region of V5/MT+: A probabilistic, stereotaxic map of area hOc5, Cereb Cortex 17 (3): 562-574, 2007

Area hOc3v [V3v]

C. Rottschy, S.B. Eickhoff, A. Schleicher, H. Mohlberg, K. Zilles, K. Amunts. The ventral visual cortex in humans: Cytoarchitectonic mapping of two extrastriate areas. Human Brain Mapping 28 (1): 1-8, 2007

Area hOc4lp

A. Malikovic, K. Amunts, A. Schleicher, H. Mohlberg, M. Kujovic, S.E. Eickhoff, K. Zilles. Cytoarchitecture of the human lateral occipital cortex: Mapping of two extrastriate areas hOc4la and hOc4lp. Brain Structure and Function, 221(4):1877-97, 2016.

Area FG1

J. Caspers, K. Zilles, S.B. Eickhoff, A. Schleicher, H. Mohlberg, K. Amunts. Cytoarchitectonical analysis and probabilistic mapping of two extrastriate areas of the human posterior fusiform gyrus. Brain Structure and Function 218: 511-526, 2013.

Cluster 2 159 vox -34 -86 -6

Assignment based on Maximum Probability Map	Percent of Cluster volume in Area	Percent of Area activated by Cluster
Area hOc4v [V4(v)]	31.4	3.7
Area hOc4la	28.7	2.7
Area FG2	11.0	2.2
Area hOc3v [V3v]	10.7	1.0
Area hOc4lp	8.8	1.0

Area FG1	0.2	0.0
Area hOc5 [V5/MT]	0.1	0.1

Probability exceedance by Area	Mean probability at Cluster location (%)	Mean probability across PMap (%)	Ratio	95% CI	95% CI
Area hOc4v [V4(v)]	33.1	25.3	1.31	1.25	1.37
Area hOc4la	28.7	30.4	0.94	0.89	0.99
Area hOc3v [V3v]	22.8	25.2	0.90	0.84	0.95
Area FG2	19.9	20.1	0.99	0.93	1.05
Area hOc4lp	19.7	25.6	0.77	0.70	0.82
Area FG1	7.9	18.9	0.42	0.39	0.45
Area hOc5 [V5/MT]	6.7	15.0	0.45	0.38	0.52
Area hOc2 [V2]	3.0	20.6	0.15	0.13	0.17
Area FG4	0.7	24.1	0.03	0.01	0.05

Top probabilites at peak voxels	Union across peaks
Area hOc4v [V4(v)]	0.9
Area hOc4la	0.9
Area FG2	0.5
Area hOc3v [V3v]	0.4
Area hOc4lp	0.4
Area FG1	0.2
Area hOc5 [V5/MT]	0.1
Area hOc2 [V2]	0.0

Probabilities at local maxima	-34.0 / -86.0 / -6.0 (Height: 7.1)	-24.0 / -86.0 / -12.0 (Height: 6.7)	-28.0 / -84.0 / -12.0 (Height: 6.7)	-40.0 / -72.0 / -8.0 (Height: 6.2)	-40.0 / -78.0 / -6.0 (Height: 6.1)	-44.0 / -80.0 / -8.0 (Height: 6.1)
Area hOc4v [V4(v)]	22.6	62.5	74.3	0.0	0.2	0.0
Area hOc4la	32.8	0.0	0.0	9.9	18.4	72.4
Area FG2	0.0	0.0	0.0	19.3	27.3	14.6

Area hOc3v [V3v]	3.3	36.4	7.0	0.0	0.0	0.0
Area hOc4lp	37.6	0.0	0.0	0.0	0.0	0.9
Area FG1	2.5	0.0	2.4	9.2	6.9	0.0
Area hOc5 [V5/MT]	0.0	0.0	0.0	0.0	2.7	5.3
Area hOc2 [V2]	0.0	1.0	0.0	0.0	0.0	0.0

Reference List

Area hOc4v [V4(v)]

C. Rottschy, S.B. Eickhoff, A. Schleicher, H. Mohlberg, K. Zilles, K. Amunts. The ventral visual cortex in humans: Cytoarchitectonic mapping of two extrastriate areas. Human Brain Mapping 28 (1): 1-8, 2007

Area FG2

J. Caspers, K. Zilles, S.B. Eickhoff, A. Schleicher, H. Mohlberg, K. Amunts. Cytoarchitectonical analysis and probabilistic mapping of two extrastriate areas of the human posterior fusiform gyrus. Brain Structure and Function 218: 511-526, 2013.

Area hOc2 [V2]

K. Amunts, A. Malikovic, H.Mohlberg, T.Schormann, K. Zilles. Brodmann's areas 17 and 18 brought into stereotaxic space - where and how variable? NeuroImage 11: 66-84, 2000

Area hOc4la

A. Malikovic, K. Amunts, A. Schleicher, H. Mohlberg, M. Kujovic, S.E. Eickhoff, K. Zilles. Cytoarchitecture of the human lateral occipital cortex: Mapping of two extrastriate areas hOc4la and hOc4lp. Brain Structure and Function, 221(4):1877-97, 2016.

Area hOc5 [V5/MT]

A. Malikovic, K. Amunts, A. Schleicher, H. Mohlberg, S.B. Eickhoff, M.Wilms, N. Palomero-Gallagher, E. Armstrong, K. Zilles. Cytoarchitectonic analysis of the human extrastriate cortex in the region of V5/MT+: A probabilistic, stereotaxic map of area hOc5, Cereb Cortex 17 (3): 562-574, 2007

Area hOc3v [V3v]

C. Rottschy, S.B. Eickhoff, A. Schleicher, H. Mohlberg, K. Zilles, K. Amunts. The ventral visual cortex in humans: Cytoarchitectonic mapping of two extrastriate areas. Human Brain Mapping 28 (1): 1-8, 2007

Area FG4

S. Lorenz, K.S. Weiner, J. Caspers, H. Mohlberg, A. Schleicher, S. Bludau, S.B. Eickhoff, K. Grill-Spector, K. Zilles, K. Amunts. Two new cytoarchitectonic areas on the human mid-fusiform gyrus. Cerebral Cortex 27 (1): 373-385, 2017.

Area hOc4lp

A. Malikovic, K. Amunts, A. Schleicher, H. Mohlberg, M. Kujovic, S.E. Eickhoff, K. Zilles. Cytoarchitecture of the human lateral occipital cortex: Mapping of two extrastriate areas hOc4la and hOc4lp. Brain Structure and Function, 221(4):1877-97, 2016.

Area FG1

J. Caspers, K. Zilles, S.B. Eickhoff, A. Schleicher, H. Mohlberg, K. Amunts. Cytoarchitectonical analysis and probabilistic mapping of two extrastriate areas of the human posterior fusiform gyrus. Brain Structure and Function 218: 511-526, 2013.

Cluster 3 3 vox +40 -60 -12

Assignment based on Maximum Probability Map	Percent of Cluster volume in Area	Percent of Area activated by Cluster
Area FG2	62.5	0.2
Area FG1	33.3	0.2

Probability exceedance by Area	Mean probability at Cluster location (%)	Mean probability across PMap (%)	Ratio	95% CI	95% CI
Area FG2	30.0	20.1	1.49	1.33	1.65
Area FG1	25.3	18.9	1.34	1.09	1.56
Area FG4	16.6	24.1	0.69	0.60	0.77
Area hOc4la	2.0	30.4	0.07	0.03	0.09
Area hOc4v [V4(v)]	0.1	25.3	0.01	0.01	0.01

Top probabilites at peak voxels		Union across peaks
Area FG1	0.3	
Area FG4	0.2	
Area FG2	0.2	

Probabilities at local maxima +40.0 / -60.0 / -12.0 (Height: 5.7)

Area FG1	27.6
Area FG4	19.0
Area FG2	16.9

Reference List

Area hOc4v [V4(v)]

C. Rottschy, S.B. Eickhoff, A. Schleicher, H. Mohlberg, K. Zilles, K. Amunts. The ventral visual cortex in humans: Cytoarchitectonic mapping of two extrastriate areas. Human Brain Mapping 28 (1): 1-8, 2007

Area FG2

J. Caspers, K. Zilles, S.B. Eickhoff, A. Schleicher, H. Mohlberg, K. Amunts. Cytoarchitectonical analysis and probabilistic mapping of two extrastriate areas of the human posterior fusiform gyrus. Brain Structure and Function 218: 511-526, 2013.

Area hOc4la

A. Malikovic, K. Amunts, A. Schleicher, H. Mohlberg, M. Kujovic, S.E. Eickhoff, K. Zilles. Cytoarchitecture of the human lateral occipital cortex: Mapping of two extrastriate areas hOc4la and hOc4lp. Brain Structure and Function, 221(4):1877-97, 2016.

Area FG4

S. Lorenz, K.S. Weiner, J. Caspers, H. Mohlberg, A. Schleicher, S. Bludau, S.B. Eickhoff, K. Grill-Spector, K. Zilles, K. Amunts. Two new cytoarchitectonic areas on the human mid-fusiform gyrus. Cerebral Cortex 27 (1): 373-385, 2017.

Area FG1

J. Caspers, K. Zilles, S.B. Eickhoff, A. Schleicher, H. Mohlberg, K. Amunts. Cytoarchitectonical analysis and probabilistic mapping of two extrastriate areas of the human posterior fusiform gyrus. Brain Structure and Function 218: 511-526, 2013.

Cluster 4 1 vox +46 -58 -12

Assignment based on Maximum Probability Map Percent of Cluster volume in Area Percent of Area activated by Cluster

Probability exceedance by Area Mean probability at Cluster location (%) Mean probability across PMap (%) Ratio 95% CI 95% CI

Area FG2	18.5	20.1	0.92	0.82	1.03
Area FG4	12.3	24.1	0.51	0.44	0.57
Area hOc4la	2.6	30.4	0.08	0.05	0.11

Top probabilites at peak voxels Union across peaks

Area FG4	0.1
Area FG2	0.1

Probabilities at local maxima +46.0 / -58.0 / -12.0 (Height: 5.7)

Area FG4	13.1
Area FG2	13.1

Reference List

Area FG2

J. Caspers, K. Zilles, S.B. Eickhoff, A. Schleicher, H. Mohlberg, K. Amunts. Cytoarchitectonical analysis and probabilistic mapping of two extrastriate areas of the human posterior fusiform gyrus. Brain Structure and Function 218: 511-526, 2013.

Area hOc4la

A. Malikovic, K. Amunts, A. Schleicher, H. Mohlberg, M. Kujovic, S.E. Eickhoff, K. Zilles. Cytoarchitecture of the human lateral occipital cortex: Mapping of two extrastriate areas hOc4la and hOc4lp. Brain Structure and Function, 221(4):1877-97, 2016.

Area FG4

S. Lorenz, K.S. Weiner, J. Caspers, H. Mohlberg, A. Schleicher, S. Bludau, S.B. Eickhoff, K. Grill-Spector, K. Zilles, K. Amunts. Two new cytoarchitectonic areas on the human mid-fusiform gyrus. Cerebral Cortex 27 (1): 373-385, 2017.

---

t-contrast Typical > Distinctive (p_{FWE}= .05)

Image: Opposite_t_Typ-Dist_FWE05 [u=0.0, k=0]

Cluster 1 8 vox -4 -64 +24

Assignment based on Maximum Probability Map Percent of Cluster volume in Area Percent of Area activated by Cluster

Probability exceedance by Area Mean probability at Cluster location (%) Mean probability across PMap (%) Ratio 95% CI 95% CI

Area 7A (SPL)1.8 24.2 0.07 0.04 0.12

Top probabilites at peak voxels Union across peaks

Area 7A (SPL)0.0

Probabilities at local maxima -4.0 / -64.0 / +24.0 (Height: 6.1)

Area 7A (SPL)0.0

Reference List

Area 7A (SPL)

F. Scheperjans, K. Hermann, S. B. Eickhoff, K. Amunts, A. Schleicher, K. Zilles. Observer-independent cytoarchitectonic mapping of the human superior parietal cortex. Cereb.Cortex 18 (4): 846-867, 2008.

F. Scheperjans, S. B. Eickhoff, L. Hömke, H. Mohlberg, K. Hermann, K. Amunts, K. Zilles. Probabilistic maps, morphometry, and variability of cytoarchitectonic areas in the human superior parietal cortex. Cereb.Cortex 18 (9): 2141-2157, 2008.

Cluster 2 4 vox -8 -46 +36

Assignment based on Maximum Probability Map Percent of Cluster volume in Area Percent of Area activated by Cluster

Probability exceedance by Area Mean probability at Cluster location (%) Mean probability across PMap (%) Ratio 95% CI 95% CI

Top probabilites at peak voxels Union across peaks

Probabilities at local maxima

Reference List

---

t-contrast High Pred Faces AND High Pred Chairs > Uninformative ($p_{unc.}$= .0001)

Image: MAIN_t_highExp-uninf_unc0001 [u=0.0, k=0]

Cluster 1 3 vox +10 +4 +26

Assignment based on Maximum Probability Map Percent of Cluster volume in Area Percent of Area activated by Cluster

Probability exceedance by Area	Mean probability at Cluster location (%)	Mean probability across PMap (%)	Ratio	95% CI	95% CI

Area 33	1.9	17.5	0.11	0.06	0.16

Top probabilites at peak voxels	Union across peaks

Probabilities at local maxima

Reference List

Area 33

Palomero-Gallagher N, Eickhoff1 SB, Hoffstaedter F, Schleicher A, Mohlberg H, Vogt BA, Amunts K, Zilles K. Functional organization of human subgenual cortical areas: relationship between architectonical segregation and connectional heterogeneity. NeuroImage 115, 177-190, 2015.

Cluster 2	1 vox	-10	-2	+30

Assignment based on Maximum Probability Map	Percent of Cluster volume in Area	Percent of Area activated by Cluster

Area 33	100.0	0.3

Probability exceedance by Area	Mean probability at Cluster location (%)	Mean probability across PMap (%)	Ratio	95% CI	95% CI

Area 33	55.2	17.5	3.15	2.80	3.51

Top probabilites at peak voxels	Union across peaks

Area 33	0.5

Probabilities at local maxima -10.0 / -2.0 / +30.0 (Height: 3.6)

Area 33	46.6

Reference List

Area 33

Palomero-Gallagher N, Eickhoff1 SB, Hoffstaedter F, Schleicher A, Mohlberg H, Vogt BA, Amunts K, Zilles K. Functional organization of human subgenual cortical areas: relationship between architectonical segregation and connectional heterogeneity. NeuroImage 115, 177-190, 2015.

F-contrast Predictability ($p_{unc.}$= .0001)

Image: MAIN_f_Pred_unc0001 [u=0.0, k=0]

Cluster 1 14 vox +10 +4 +26

Assignment based on Maximum Probability Map Percent of Cluster volume in Area Percent of Area activated by Cluster

Probability exceedance by Area Mean probability at Cluster location (%) Mean probability across PMap (%) Ratio 95% CI 95% CI

Area 33	5.3	17.5	0.30	0.21	0.40

Top probabilites at peak voxels Union across peaks

Probabilities at local maxima

Reference List

Area 33

Palomero-Gallagher N, Eickhoff1 SB, Hoffstaedter F, Schleicher A, Mohlberg H, Vogt BA, Amunts K, Zilles K. Functional organization of human subgenual cortical areas: relationship between architectonical segregation and connectional heterogeneity. NeuroImage 115, 177-190, 2015.

Cluster 2 12 vox -10 -2 +30

Assignment based on Maximum Probability Map Percent of Cluster volume in Area Percent of Area activated by Cluster

Area 33 50.0 1.6

Probability exceedance by Area Mean probability at Cluster location (%) Mean probability across PMap (%) Ratio 95% CI 95% CI

Area 33 38.5 17.5 2.20 1.96 2.45

Top probabilites at peak voxels Union across peaks

Area 33 0.5

Probabilities at local maxima -10.0 / -2.0 / +30.0 (Height: 18)

Area 33 46.6

Reference List

Area 33

Palomero-Gallagher N, Eickhoff1 SB, Hoffstaedter F, Schleicher A, Mohlberg H, Vogt BA, Amunts K, Zilles K. Functional organization of human subgenual cortical areas: relationship between architectonical segregation and connectional heterogeneity. NeuroImage 115, 177-190, 2015.

Cluster 3 1 vox -40 -28 -2

Assignment based on Maximum Probability Map Percent of Cluster volume in Area Percent of Area activated by Cluster

Probability exceedance by Area	Mean probability at Cluster location (%)	Mean probability across PMap (%)	Ratio	95% CI	95% CI
Area Id1	7.9	15.9	0.50	0.20	0.79
Area Ig1	0.7	15.4	0.05	0.02	0.08

Top probabilites at peak voxels	Union across peaks
Area Id1	0.2
Area Ig1	0.0

Probabilities at local maxima -40.0 / -28.0 / -2.0 (Height: 13)

Area Id1	19.2
Area Ig1	0.3

Reference List

Area Id1

F. Kurth, S. B. Eickhoff, A. Schleicher, L. Hoemke, K. Zilles, and K. Amunts. Cytoarchitecture and probabilistic maps of the human posterior insular cortex. Cereb.Cortex 20 (6):1448-1461, 2009.

Area Ig1

F. Kurth, S. B. Eickhoff, A. Schleicher, L. Hoemke, K. Zilles, and K. Amunts. Cytoarchitecture and probabilistic maps of the human posterior insular cortex. Cereb.Cortex 20 (6):1448-1461, 2009.

F-contrast Predictability x Typicality Interaction ($p_{unc.}$= .0001)

No significant results
